# PUMAS: fine-tuning polygenic risk scores with GWAS summary statistics

**DOI:** 10.1186/s13059-021-02479-9

**Published:** 2021-09-06

**Authors:** Zijie Zhao, Yanyao Yi, Jie Song, Yuchang Wu, Xiaoyuan Zhong, Yupei Lin, Timothy J. Hohman, Jason Fletcher, Qiongshi Lu

**Affiliations:** 1 Department of Biostatistics and Medical Informatics, University of Wisconsin-Madison, Madison, WI 53703 USA; 2 Department of Statistics, University of Wisconsin-Madison, Madison, WI USA; 3 University of Wisconsin-Madison, Madison, WI USA; 4 Vanderbilt Memory and Alzheimer’s Center, Vanderbilt University Medical Center, Vanderbilt University School of Medicine, Nashville, TN USA; 5 Vanderbilt Genetics Institute, Vanderbilt University Medical Center, Nashville, TN USA; 6 La Follette School of Public Affairs, University of Wisconsin-Madison, Madison, WI USA; 7 Department of Sociology, University of Wisconsin-Madison, Madison, WI USA; 8 Center for Demography of Health and Aging, University of Wisconsin-Madison, Madison, WI USA

**Keywords:** GWAS, Polygenic risk score, Model tuning, Summary statistics

## Abstract

Polygenic risk scores (PRSs) have wide applications in human genetics research, but often include tuning parameters which are difficult to optimize in practice due to limited access to individual-level data. Here, we introduce PUMAS, a novel method to fine-tune PRS models using summary statistics from genome-wide association studies (GWASs). Through extensive simulations, external validations, and analysis of 65 traits, we demonstrate that PUMAS can perform various model-tuning procedures using GWAS summary statistics and effectively benchmark and optimize PRS models under diverse genetic architecture. Furthermore, we show that fine-tuned PRSs will significantly improve statistical power in downstream association analysis.

**Supplementary Information:**

The online version contains supplementary material available at 10.1186/s13059-021-02479-9.

## Background

Accurate prediction of complex traits with genetic data is a major goal in human genetics research and precision medicine [1]. In the past decade, advancements in genotyping and imputation techniques have greatly accelerated discoveries in genome-wide association studies (GWASs) for numerous complex diseases and traits [2]. These data have also enabled statistical learning applications that leverage genome-wide data in genetic risk prediction [3–8]. However, despite these advances, it remains challenging to access, store, and process individual-level genetic data at a large scale due to privacy concerns and high computational burden. With increasingly accessible GWAS summary statistics for a variety of complex traits [9], polygenic risk scores (PRSs) that use marginal association statistics as input enjoy great popularity and have had success in diverse applications [10–12].

With great popularity, there also come great challenges. Prediction accuracy of PRS remains moderate for most phenotypes [13]. Methods have been developed to improve PRS performance by explicitly modeling linkage disequilibrium (LD) [14], incorporating functional annotations and pleiotropy [15, 16], and improving effect estimates through statistical shrinkage [17]. Notably, most PRS models have tuning parameters, including the *p*-value threshold in traditional PRS, the penalty strength in penalized regression models, and the proportion of causal variants in LDpred [14]. Tuning parameters are very common in predictive modeling. When properly selected, these parameters add flexibility to the model and improve prediction accuracy. This is a well-understood problem with a rich literature—a well-known solution is cross-validation [18]. However, most model-tuning methods require individual-level genetic data either as the training dataset or as a validation dataset independent from both the input GWAS and the testing samples. In practice, these data rarely exist, especially when PRS is generated using GWAS summary statistics in the public domain. This has created a significant gap between current conventions in PRS construction and optimal methodologies. Without a method to fine-tune models using summary statistics, it is challenging to benchmark and optimize PRS, thus limiting its clinical utility.

We introduce PUMAS (Parameter-tuning Using Marginal Association Statistics), a novel method to fine-tune PRS models using GWAS summary data. As a general framework, PUMAS can conduct a variety of model-tuning procedures on PRS, including training-testing data split, cross-validation, and repeated learning. Through extensive simulations on realistic genetic architecture, we demonstrate that the performance of PUMAS is as good as methods based on individual-level data. Additionally, we apply PUMAS to GWAS traits with distinct types of genetic architecture and validate our results using well-powered external datasets. Furthermore, we systematically benchmark and optimize PRS for numerous diseases and traits and showcase the immediate benefits of fine-tuned PRSs in downstream applications.

## Results

### Method overview

**Fig. 1:**
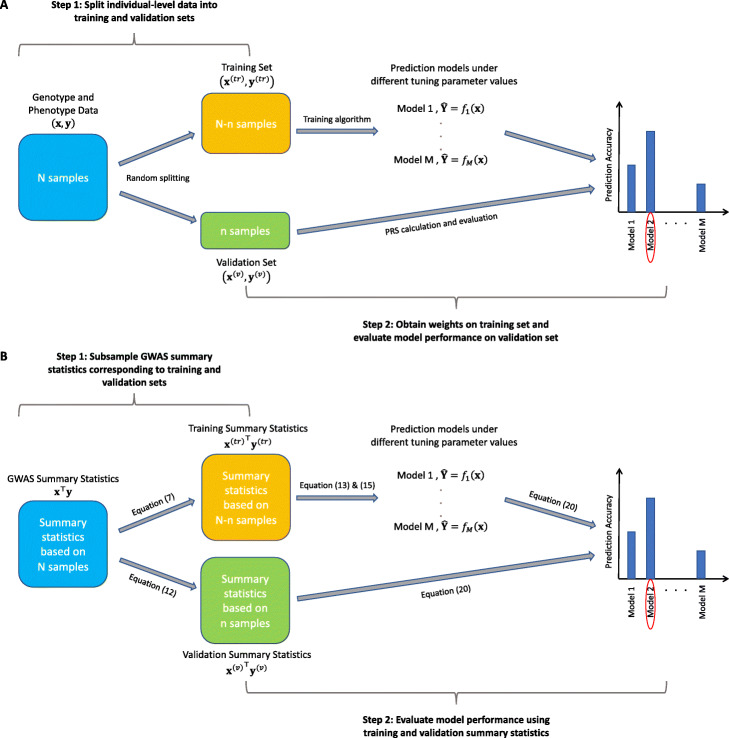
A workflow of model-tuning strategies. **A** Traditional approaches split individual-level data into training and validation subsets to fine-tune prediction models. **B** Our method directly generates training and validation summary statistics without using individual-level information and use simulated summary statistics as input to select the best model

Here, we outline the PUMAS framework. Detailed derivations and technical discussions are included in the “Methods” section. There are two key steps in our proposed model-tuning framework (Fig. 1). First, we sample marginal association statistics for a subset of individuals based on the complete GWAS summary data (Eqs. (7) and (12), the “Methods” section). Using this approach, we can generate summary statistics for independent training and validation sets without actually partitioning the samples. Second, we propose an approach to evaluate the predictive performance (e.g., predictive *R*^2^) of PRS using summary statistics in the validation set so that we can select the best model based on its superior performance (Eq. (20), the “Methods” section). These two steps together make it possible to select the best-performing model with only one set of GWAS summary statistics as input.

### Simulation results

**Fig. 2:**
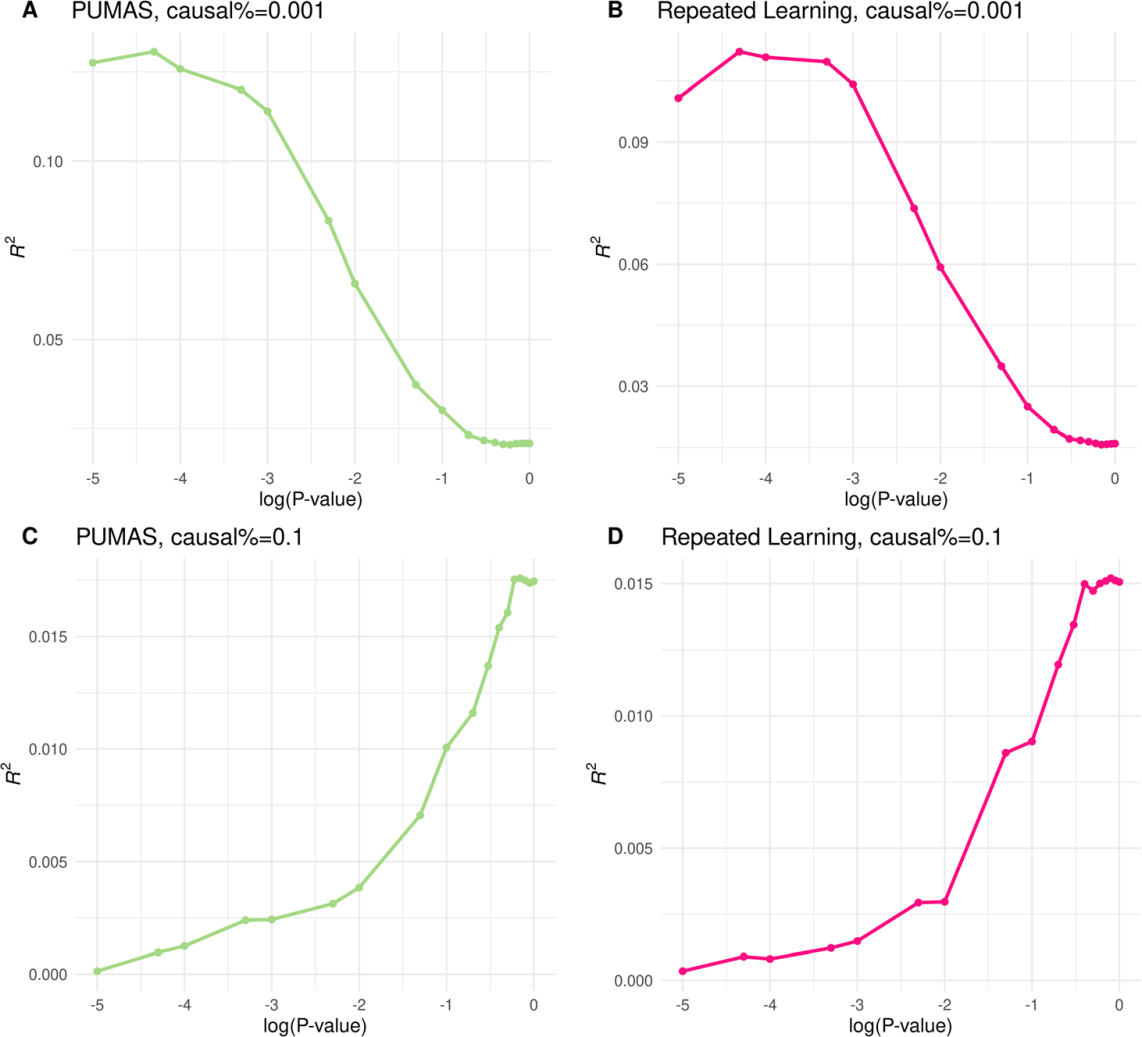
Comparison of PUMAS and repeated learning. **A**, **C** Model tuning results based on PUMAS. **B**, **D** Results of repeated learning with individual-level data as input. The proportion of causal variants was set to be 0.001 in **A** and **B** and 0.1 in **C** and **D**. The *X*-axis shows the log-transformed *p*-value thresholds. The *Y*-axis shows the predictive performance quantified by average *R*^2^ across four folds. Parameter *α* was set to be 0 in this simulation (the “Methods” section). Results for other settings are summarized in Additional file 1: Fig. S1-S9

We conducted simulations using genotype data from the Wellcome Trust Case Control Consortium (WTCCC) to investigate if PUMAS can achieve similar performance compared to classic model-tuning procedures. A total of 15,567 individuals and 322,235 genetic variants were included in the simulation after quality control. We simulated phenotype data with a heritability of 0.5, varying assumptions on SNP effects and proportion of causal variants (the “Methods” section). We used these data to calculate marginal association statistics and ranked SNPs based on association *p*-values. Next, we applied PUMAS to perform 4-fold repeated learning on marginal association statistics and selected the optimal number of SNPs to include in the prediction model by maximizing the average *R*^2^ across folds. Additionally, we implemented a traditional repeated learning approach with the same simulated individual-level data as a reference. Details about our implementation of PUMAS and repeated learning are described in the “Methods” section. Overall, these two approaches yielded equivalent results on both quantitative and binary traits (Fig. 2 and Additional file 1: Fig. S1-S9; Additional file 2 and 3: Table. S1-S2). Across all simulation settings, our summary statistics-based approach showed nearly identical results compared to a state-of-the-art model-tuning approach based on individual-level data and could effectively select the optimal tuning parameter (i.e., number of SNPs in the PRS).

### PUMAS effectively fine-tunes PRS models based on genetic architecture

Next, we demonstrate our method’s performance using a gold-standard approach—we apply PUMAS to the summary statistics from well-powered GWASs to select the optimal *p*-value cutoffs in PRS models and validate their performance on large independent cohorts. First, we applied PUMAS to a recent GWAS of educational attainment (EA) conducted by the Social Science Genetic Association Consortium (*N* = 742,903) [19]. 4775 samples with European ancestry in the National Longitudinal Study of Adolescent to Adult Health (Add Health) [20] and 10,214 European samples in the Health and Retirement Study (HRS) [21] were used as two independent validation sets to assess the predictive performance of EA PRS. We used GWAS of Alzheimer’s disease (AD) as a second example. We applied PUMAS to the stage-1 summary statistics from the 2013 study conducted by the International Genomics of Alzheimer’s Project (IGAP; *N* = 54,162) [22] to optimize PRS models for AD. These PRSs were then evaluated on summary-level data of 7050 independent samples [23] from the Alzheimer’s Disease Genetics Consortium (ADGC) and individual-level data of 355,583 samples in the UK Biobank with a family history-based proxy phenotype for AD (the “Methods” section) [24].

**Fig. 3:**
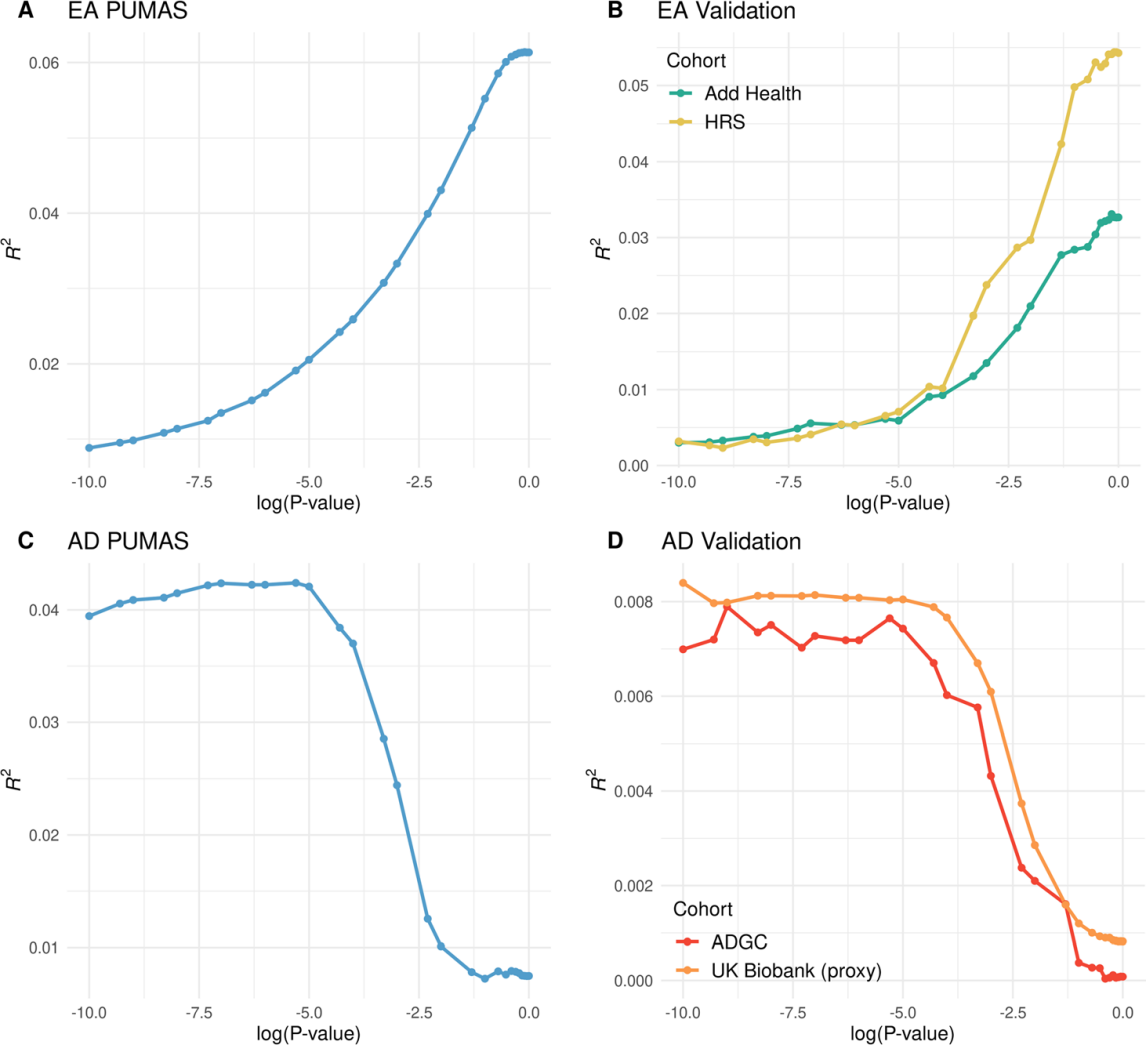
Model-tuning performance on real GWAS data. **A** PUMAS performance on the EA training set. **B** Prediction performance on two validation sets for EA. **C** PUMAS performance on the AD training set. **D** Prediction performance on two validation sets for AD. The *X*-axis shows the log-transformed *p*-value cutoffs in PRS which is the tuning parameter of interest. The *Y*-axis indicates predictive *R*^2^. EA educational attainment, AD Alzheimer’s disease

Our summary statistics-based analyses showed highly consistent results compared with external validations (Fig. 3; Additional file 4: Table. S3). Our analysis clearly suggested that a model with a large number of SNPs tends to be more predictive for EA, a pattern validated in both Add Health and HRS cohorts. The EA PRS based on *p*-value cutoffs of 0.8, 0.8, and 0.7 were the most predictive models suggested by PUMAS, HRS, and Add Health cohorts, respectively. Results on AD were also consistent between PUMAS and external validations. The optimal *p*-value cutoffs suggested by PUMAS, ADGC validation, and UK Biobank validation were 5e−6, 1e−9, and 1e−10, respectively. PRS models based on *p*-value cutoffs more stringent than 1e−5 showed good predictive performance in two validation sets for AD. Notably, as more SNPs are included in the model, the predictive performance of PRS sharply declines. Our model-tuning results based on GWAS summary statistics accurately predicted this pattern. Additionally, since we used an AD-proxy phenotype in the UK Biobank, the reduced predictive *R*^2^ is expected. But the trend of predictive performance remained consistent with the validation result in case-control data from the ADGC.

EA is known to be extremely polygenic—more than 1200 independent genetic associations have been identified for EA to date [19]. AD has a very different genetic architecture compared to EA. The *APOE* locus has an unusually large effect on AD risk [25]. In addition to *APOE*, about 30 independent loci have been implicated in AD GWASs [26]. Our method correctly suggested that the EA PRS would perform better if more SNPs are in the model (87,985 SNPs were included) while a substantially sparser model with 31 SNPs would yield better predictive performance for AD. These results showcased our method’s ability to adaptively choose the optimal tuning parameter for traits with different patterns of genetic architecture. These results also highlighted the importance of model tuning. An AD PRS based on an arbitrary *p*-value cutoff of 0.01 can have a 5-fold reduction in predictive *R*^2^ compared to the fine-tuned PRS.

### Some technical considerations

**Fig. 4:**
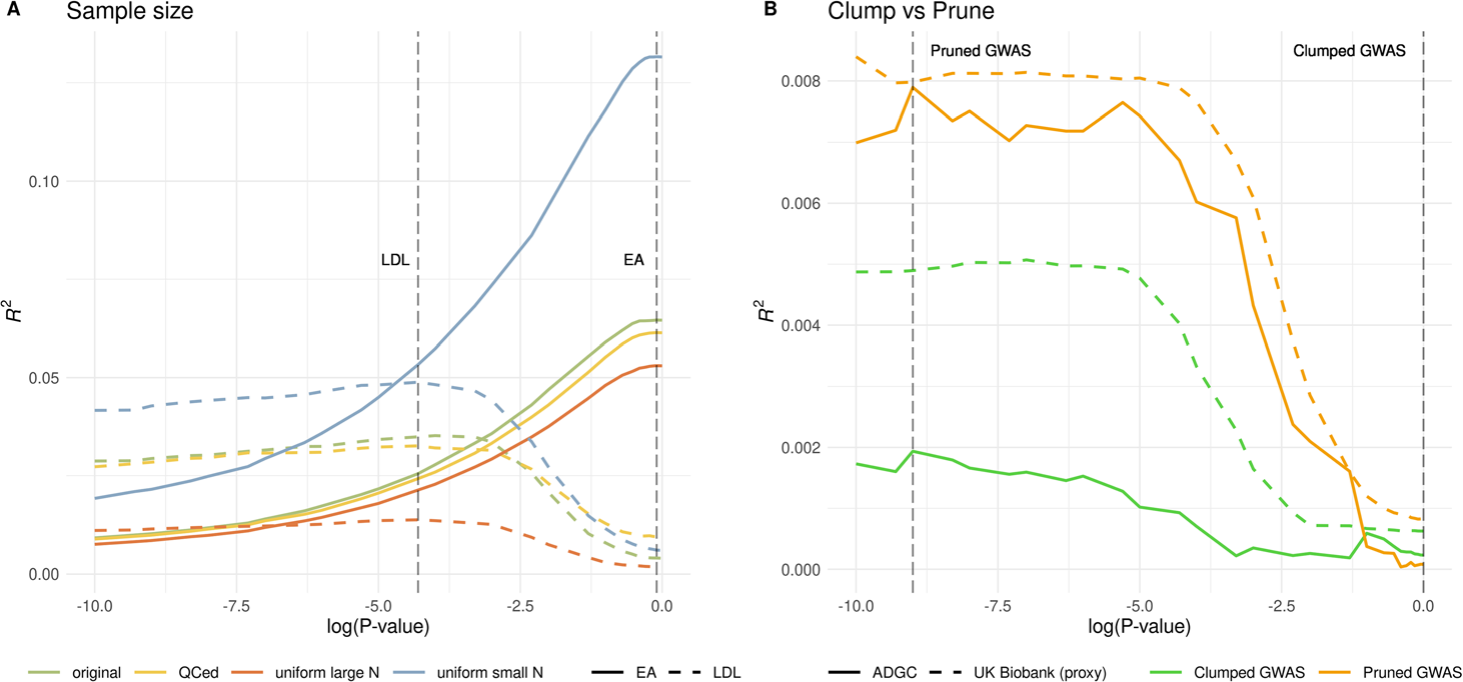
Technical issues involving sample size and LD clumping. **A** PUMAS results on LDL cholesterol and EA with various sample size specifications. The two gray dashed lines represent the optimal *p*-value cutoffs selected by the “QCed” setting for LDL and EA, respectively. **B** Predictive performance on external validation for AD PRS based on pruned and clumped summary statistics. Two gray dashed lines mark the optimal *p*-value cutoffs inferred by PUMAS on pruned and clumped summary statistics. LDL low-density lipoprotein, EA educational attainment, AD Alzheimer’s disease

We discuss two unique technical issues that may arise in summary statistics-based model tuning. First, sample sizes for different SNPs in a GWAS meta-analysis may vary due to technical differences across cohorts. However, it is not uncommon for a GWAS to only report the maximum sample size. Here, we investigate the robustness of PUMAS when the sample size is mis-specified. We use two GWAS datasets that provided accurate sample size for each SNP: summary statistics for low-density lipoprotein (LDL) cholesterol from the Global Lipids Genetics Consortium (GLGC; *N* = 188,577) [27] and the same EA GWAS summary statistics we have described before. We compared PUMAS results based on four different approaches. The first approach uses the accurate sample size reported in the summary statistics (“original”). The second approach removes SNPs with sample size below the 30% quantile of its distribution and uses the accurate sample size for the remaining SNPs (“QCed”). The third and fourth approaches apply the maximum or minimum sample size to all SNPs (“Uniform large/small N”). For the “original” and “QCed” approaches where precise sample size is available for each SNP, we assigned 25% of the minimal *N* value as the sample size for the validation dataset and used the remaining samples of each SNP in the training subset. Overall, PUMAS results showed consistent patterns under these four scenarios (Fig. 4A; Additional file 5: Table. S4). Although the *R*^2^ estimates can inflate or deflate if the sample size is mis-specified, the optimal *p*-value cutoffs selected by PUMAS remained stable. Thus, PUMAS can still select the best-performing model even if accurate sample size information is unavailable. In practice, performing quality control to remove SNPs with outlier sample size may make the *R*^2^ estimates most interpretable.

The second issue is to see if PUMAS can be applied to clumped GWAS summary statistics. In PRS applications, it is a common practice to clump the data by removing SNPs in strong LD with the most significant SNP in a region. However, since *p*-values based on the full sample have been used during LD clumping, directly applying the same model-tuning methods to clumped data may lead to information leak and overfitting. We applied PUMAS to clumped summary statistics of the IGAP 2013 AD GWAS (Additional file 1: Fig. S10). The model-tuning results in PUMAS were completely inconsistent with the optimal models in external validation (Fig. 4B; Additional file 6: Table. S5), confirming that PUMAS should not be applied to clumped data. However, we note that the predictive curves were very similar in external validations no matter if pruned or clumped data were used as input. Therefore, in practice, it may be plausible to apply PUMAS to pruned GWAS summary data and obtain the optimal *p*-value threshold. This way, *p*-values based on the complete sample will not influence the model-tuning procedure. Then, we can apply this selected *p*-value cutoff with clumped GWAS summary statistics to calculate PRS.

### Benchmarking and optimizing PRS for 65 diseases and traits

**Fig. 5:**
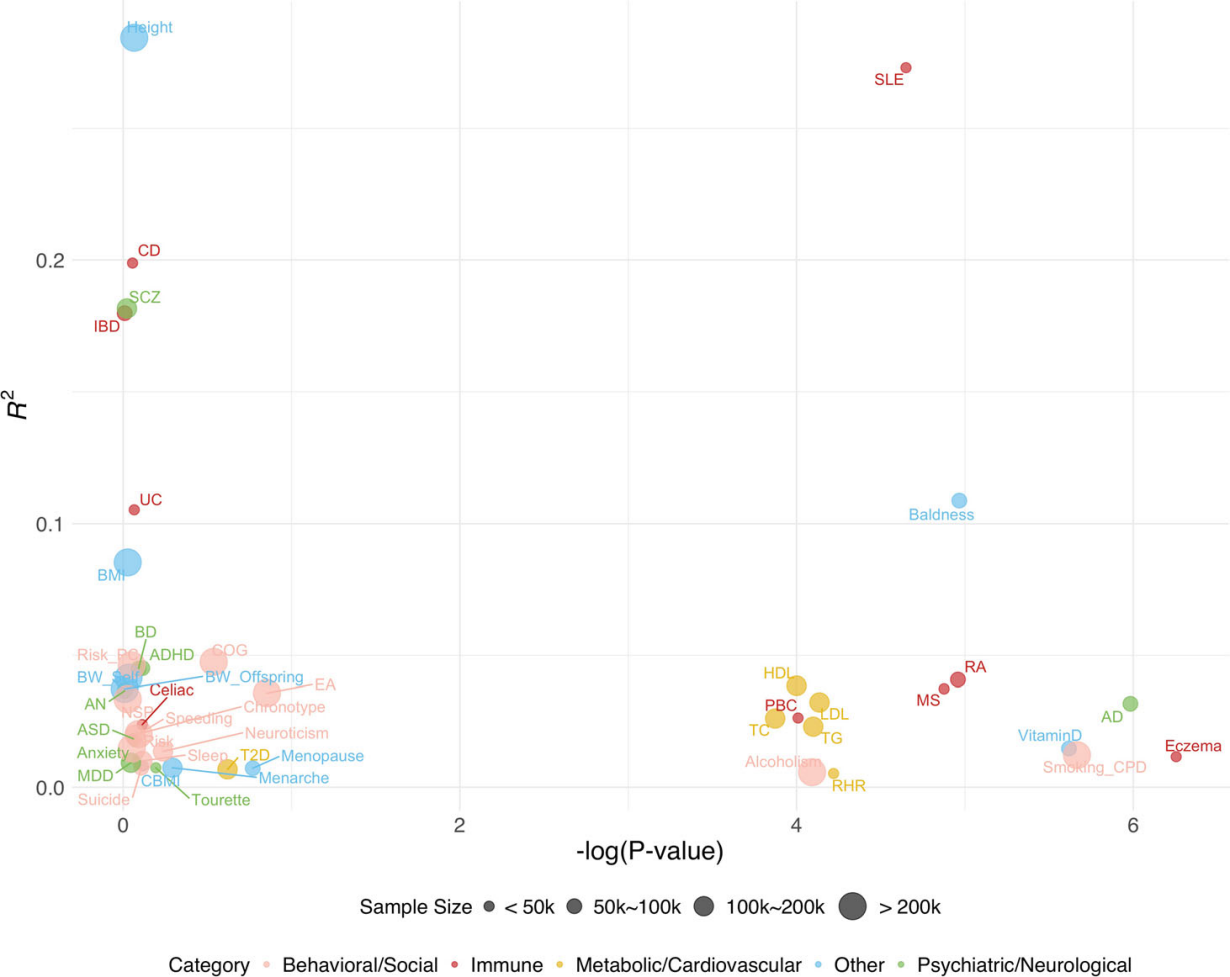
An atlas of optimized PRSs for complex diseases and traits. 45 diseases/traits with optimized *R*^2^ > 0.005 are included in the figure. Each circle represents a disease or trait. The size of circles indicates the sample size of the study; colors mark the five trait categories. The *X*-axis indicates the negative log-transformed *p*-value cutoff in PRS which is also the tuning parameter of interest. The *Y*-axis indicates the optimal *R*^2^. Information on all diseases and traits is summarized in Additional file 7: Table. S6

Next, we apply PUMAS to provide an atlas of optimized PRSs for complex diseases and traits (Fig. 5). In total, we analyzed 65 GWASs with available summary statistics and documented each trait’s optimal *p*-value cutoff and predictive *R*^2^ (Additional file 7: Table. S6). The average gain in predictive *R*^2^ with our method is 0.0106 (205.6% improvement) and 0.0034 (62.5% improvement) compared to PRSs with *p*-value cutoffs of 0.01 and 1, respectively (Additional file 1: Fig. S11 and Additional file 8: Table. S7). We annotated the traits into five categories: behavioral/social, metabolic/cardiovascular, psychiatric/neurological, immune, and others. Most behavioral/social traits and psychiatric/neurological disorders had optimal *p*-value cutoffs between 0.1 and 1 which is consistent with their extreme polygenic genetic architecture. The exceptions include alcoholism (drinks per week), smoking behavior (cigarettes per day), and AD. PRSs with fewer SNPs showed superior performance for these traits. Among immune diseases, systemic lupus erythematosus, primary biliary cirrhosis, rheumatoid arthritis, multiple sclerosis, and eczema all favored a sparse model, while the optimal PRSs for inflammatory bowel diseases and celiac disease had substantially more SNPs. We also note that molecular traits such as blood lipids and 25-hydorxyvitamin D favored sparse PRS models, possibly due to stronger genetic effects and more homogeneous genetic mechanisms. These results also shed light on the differences in the predictive power of diverse types of diseases and traits. PRSs for height, systemic lupus erythematosus, inflammatory bowel diseases, and schizophrenia showed substantially better predictive performance, while the *R*^2^ for most behavioral/social traits remained moderate despite the large sample size in those studies. We also investigated the computational efficiency of our approach in real GWAS applications. Using only one CPU, PUMAS has an average computation time of 8.3 s and maximum of 38.23 s in the analyses of 65 traits (Additional file 1: Fig. S12; Additional file 9: Table. S8), showing computationally scalable performance.

### Identifying neuroimaging associations for AD

Finally, we demonstrate that fine-tuned PRS will lead to power gain in association analysis. We generated PRSs for 211 neuroimaging traits based on two recent studies conducted using samples from the UK Biobank (*N* = 17,706 and 19,629 for diffusion tensor imaging traits and regional volume phenotypes, respectively) [28, 29]. We optimized PRS for each imaging trait using PUMAS (Additional file 10: Table. S9). For comparison, we also generated PRSs for all traits using an arbitrary *p*-value cutoff of 0.01. We applied the BADGERS [30] approach to test associations between 211 neuroimaging trait PRSs with AD in two large, independent AD datasets: the 2019 IGAP GWAS for AD (*N* = 63,926) [26] and the UK Biobank-based GWAS with a proxy phenotype for AD (*N* = 318,773) [24, 26]. Samples used in the neuroimaging GWAS were removed from the AD-proxy GWAS to avoid overfitting of PRS models (the “Methods” section; Additional file 1: Fig. S13 and Additional file 11: Table. S10). Association results in two AD datasets were meta-analyzed to improve statistical power.

**Fig. 6:**
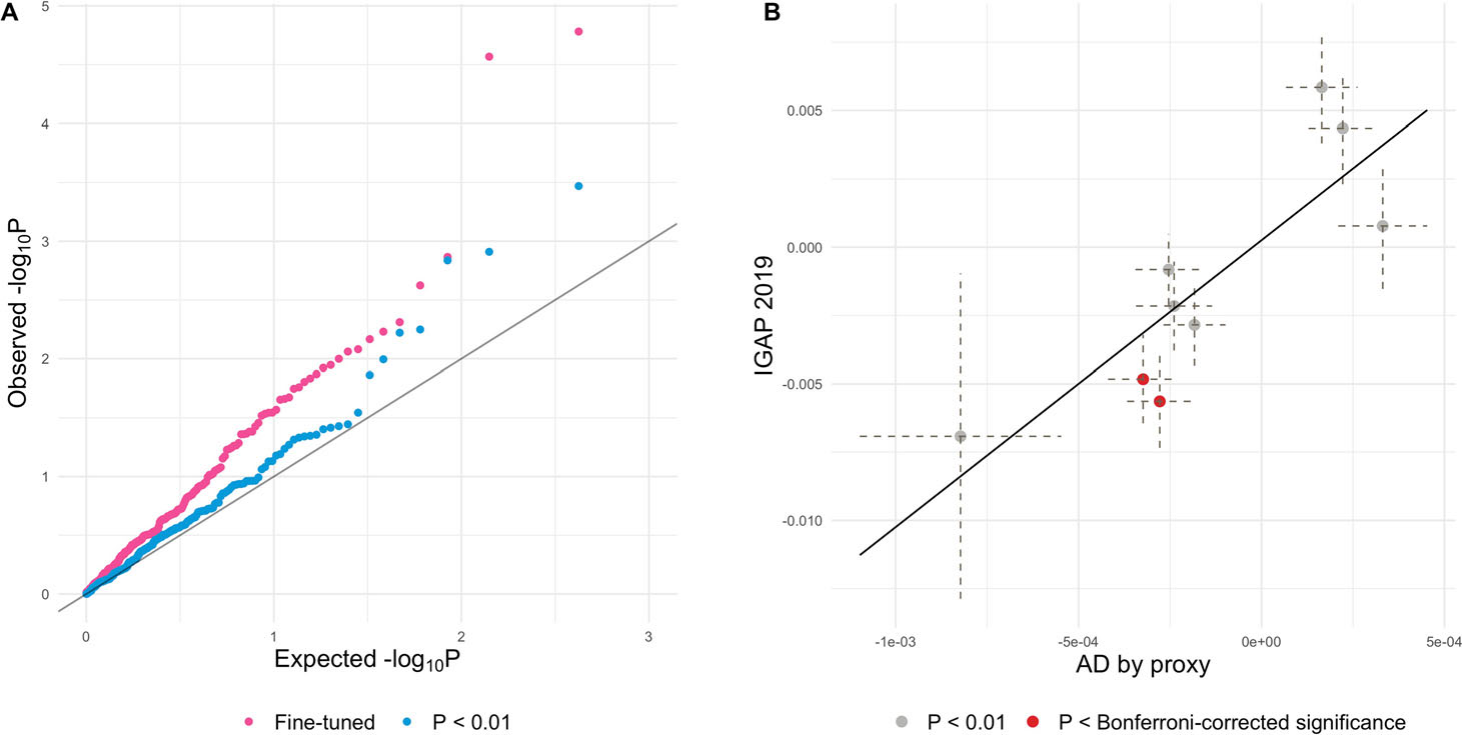
Identifying neuroimaging trait PRSs associated with AD. **A** QQ plot for the associations between 211 neuroimaging trait PRSs and AD. *p*-values were based on the meta-analysis of IGAP 2019 GWAS and the UK Biobank with a proxy AD phenotype. **B** Effect size estimates for top associations. Imaging trait PRSs that reached a *p*-value < 0.01 in the meta-analysis are shown in the plot. *X*-axis: effect sizes of imaging trait PRSs on the AD-proxy phenotype in the UK Biobank; *Y*-axis: effect sizes on AD in the IGAP 2019 GWAS. Imaging traits whose *p*-value achieved Bonferroni-corrected significance in the meta-analysis are highlighted in red. The dashed lines represent the standard error of effect size estimates

The complete association results of 211 neuroimaging traits with AD are summarized in Additional file 12: Table. S11. Using fine-tuned PRSs, we identified 2 significant associations with AD under a stringent Bonferroni correction for multiple testing: fornix (cres)/stria terminalis mode of anisotropy (*p* = 1.7E−05) and axial diffusivities (*p* = 2.7E−05) whereby genetic risk for worse white matter integrity in the fornix was associated with risk of AD. No significant associations were identified using PRSs with an arbitrary *p*-value cutoff (Fig. 6A). Association *p*-values based on optimized PRSs were significantly lower than those based on arbitrary PRSs (*p* = 0.03; two-sample Kolmogorov-Smirnov test). Additionally, effect size estimates for top associations were consistent in two independent AD GWASs (Fig. 6B). Although the effect sizes in two AD studies were not at the same scale due to the difference in AD phenotype definition, effect estimates showed strong concordance between two independent analyses (correlation = 0.84). The fornix is a critical white matter tract projecting from the medial temporal lobe where pathology begins in AD; thus, it is unsurprising that microstructural changes in the fornix measured with diffusion tensor imaging are observed in mild cognitive impairment and AD [31–33]. Furthermore, as a negative control, we applied the same analysis to a well-powered breast cancer GWAS (*N* = 228,951) [34]. Results for fine-tuned PRSs and arbitrary PRSs were consistent with the expectation under the null (Additional file 1: Fig. S14). No significant associations were identified. These findings demonstrated that our model-tuning approach can increase the statistical power in PRS association analysis.

## Discussion

Fine-tuning PRS models with GWAS summary statistics has long been considered an impossible task. In this work, we introduced a statistical framework to solve this challenging problem. First, using GWAS summary data as input, PUMAS simulates training and validation summary statistics without accessing individual-level information. Then, PUMAS evaluates and optimizes PRS models on the simulated validation summary statistics. Both steps in the PUMAS framework are statistically rigorous, computationally efficient, and highly novel. Through simulations and analysis of real GWAS data with diverse genetic architecture, we demonstrated that PUMAS can effectively conduct sophisticated model-tuning tasks using GWAS summary statistics. We also showed that optimizing PRSs improves the statistical power in downstream association analysis and identified neuroimaging traits significantly associated with AD.

This work will bring multiple advances to the field. First, it is no longer necessary to leave one dataset out in the GWAS for model-tuning purpose. With PUMAS, researchers can safely use effect size estimates from the largest available GWAS for PRS model training, which will lead to improved prediction accuracy. Second, when an independent validation set is not available, most studies in the literature select tuning parameters using one of the two strategies. Some studies fine-tune PRSs on testing samples that are used again in downstream applications, creating an overfitting problem, while other studies use a subset of testing samples to tune the model, reducing the sample size and power in the testing data. PUMAS allows researchers to apply fine-tuned PRS models to the full testing samples, thus avoiding overfitting and improving statistical power. Third, selecting the optimal tuning parameter is not the only application of PUMAS. Given a PRS model, our method allows researchers to calculate cross-validated predictive accuracy, providing a systematic approach to benchmark model performance without requiring external samples.

Our proposed framework has some limitations. First, our analyses so far have only focused on a classic PRS model with pruned SNPs and a varying *p*-value cutoff that needs to be tuned. Despite the simplicity, it remains one of the most widely used PRS models in the field. However, more sophisticated PRS methods have emerged [35–39]. Future work will focus on generalizing PUMAS to fine-tune parameters in other PRS models and benchmarking the performance of all models for different traits. Second, although we demonstrated that PUMAS can also select the optimal tuning parameters for PRS of binary traits, the approximated *R*^2^ metric is less interpretable. A future direction is to explore other metrics (e.g., AUC) to quantify prediction performance for binary traits. Third, our method assumes that GWAS is performed on independent samples. It is an open question whether PUMAS can be directly applied to family-based GWAS results [40–43].

Our results have provided strong evidence that it is possible to fine-tune PRS models with GWAS summary data. This new approach, in conjunction with widely available GWAS summary statistics, will have a long-lasting impact on future PRS model development and genetic prediction applications.

## Methods

### Step 1: Subsampling GWAS association statistics

#### Step 1-a: Specify sampling distribution of summary statistics

We assume the quantitative trait *Y* follows a linear model:

1 $$ Y=\mathbf{X}\boldsymbol{\upbeta } +\epsilon $$

where **X** ***=*** (*X*_1_, …, *X*_*p*_) denotes the random vector of *p* SNP; **β** ***=*** (β_1_, …, β_*p*_)^***T***^ is a *p*-dimensional vector representing fixed SNP effect sizes; *ϵ* is the error term following a normal distribution with zero mean. Let **y** and **x** = (**x**_1_, …, **x**_*p*_) denote the observed *N* × 1 phenotypic and *N* × *p* genotypic data of *N* independent individuals. For simplicity, we assume **y** and **x**_*j*_’s are centered. The summary association statistics in GWAS are obtained from the marginal linear regressions. Then, for *j* = 1, …, *p*, we can denote the regression coefficients and their standard errors as follows:

2 $$ {\hat{\beta}}_j={\left({\mathbf{x}}_j^{\mathrm{T}}{\mathbf{x}}_j\right)}^{-1}\left({\mathbf{x}}_j^{\mathrm{T}}\mathbf{y}\right) $$

3 $$ \mathrm{SE}\left({\hat{\beta}}_j\right)=\sqrt{\frac{{\hat{\varepsilon_j}}^{\top}\hat{\varepsilon_j}}{\left(N-1\right){\mathbf{x}}_j^{\top }{\mathbf{x}}_j}} $$

where ϵ_*j*_ is the error term for the marginal linear regression of phenotype on the *j*th SNP and $\hat{\varepsilon_j}=\mathbf{y}-{\mathbf{x}}_j{\hat{\beta}}_j$ is the observed residual from the *j*th marginal regression. If we have access to the full data set, most model-tuning approaches involve randomly sampling a subset of *N* − *n* individuals as the training set, i.e., **y**^(*tr*)^ and **x**^(*tr*)^. Naturally, the remaining subset of *n* individuals will be the validation dataset denoted as **y**^(*v*)^ and **x**^(*v*)^. When only the summary statistics file based on the full dataset is provided, the traditional model-tuning approaches cannot be implemented. Instead, we propose a method to generate marginal summary statistics for the training and validating datasets from summary statistics of the full dataset. By central limit theorem, as sample size *N* → ∞, we have

4 $$ {\mathbf{x}}^{\mathrm{T}}\mathbf{y}\sim \mathbf{N}\left(N\mathrm{E}\left({\mathbf{X}}^{\mathrm{T}}Y\right),N\mathrm{Var}\left({\mathbf{X}}^{\mathrm{T}}Y\right)\right) $$

5 $$ {\mathbf{x}}^{(tr)^{\mathrm{T}}}{\mathbf{y}}^{(tr)}\sim \mathbf{N}\left(\left(N-n\right)\mathrm{E}\left({\mathbf{X}}^TY\right),\left(N-n\right)\mathrm{Var}\left({\mathbf{X}}^TY\right)\right) $$

6 $$ {\mathbf{x}}^{(v)^{\mathrm{T}}}{\mathbf{y}}^{(v)}\sim \mathbf{N}\left(n\mathrm{E}\left({\mathbf{X}}^TY\right),n\mathrm{Var}\left({\mathbf{X}}^TY\right)\right) $$

where **x**^*T*^**y** is the observed *p* × 1 summary statistics for *N* individuals and *p* genetic markers that can be directly calculated or approximated from the full GWAS summary statistics, and **x**^(*tr*)⊤^**y**^(*tr*)^ and **x**^(*v*)⊤^**y**^(*v*)^ represent summary statistics for two partitions of full GWAS samples, which are the training set and validation set, respectively. In the following derivation, we use superscripts (*tr*) and (*v*) to indicate whether any summary statistics are computed from **x**^(*tr*)⊤^**y**^(*tr*)^ or **x**^(*v*)⊤^**y**^(*v*)^. It can be shown that

7 $$ {\mathbf{x}}^{(tr)^{\mathrm{T}}}{\mathbf{y}}^{(tr)}\mid {\mathbf{x}}^{\mathrm{T}}\mathbf{y}\sim \mathbf{N}\left(\frac{\left(N-n\right)}{N}{\mathbf{x}}^{\mathrm{T}}\mathbf{y},\frac{\left(N-n\right)}{N}\boldsymbol{\Sigma} \right)\operatorname{} $$

where · ∣ · denotes the conditional distribution and **Σ** is the observed covariance matrix of **x**^⊤^**y** from the GWAS data. A detailed derivation of this conditional distribution is included in Additional file 1. Note that until now our framework does not depend on the assumption of linkage equilibrium.

#### Step 1-b: Estimate covariance matrix of summary statistics

To subsample summary statistics, we now estimate the covariance matrix of **x**^⊤^**y**. Under simple scenarios where the SNPs are independent (i.e., GWAS summary statistics is pruned), **Σ** is a symmetric matrix whose diagonal and non-diagonal elements can be denoted as

8 $$ {\Sigma}_j={\beta}_j^2\mathrm{Var}\left({X}_j^2\right)+E\left({\epsilon}_j^2\right)E\left({X}_j^2\right) $$

9 $$ {\Sigma}_{ji}={\beta}_j{\beta}_iE\left({X}_j^2\right)E\left({X}_i^2\right) $$

For *j* = 1, …, *p* and *i* ≠ *j*. Here, $E\left({\epsilon}_j^2\right)$ can be estimated by the mean squared error in marginal regressions, which can be further approximated by $N{\left[\mathrm{SE}\left(\hat{\beta_j}\right)\right]}^2E\left({X}_j^2\right)$. In addition, each SNP’s effect size (i.e., *β*_*j*_) is typically very small in GWAS and $E\left({X}_j^2\right)$ only depends on each SNP’s minor allele frequency (MAF) which is commonly provided in GWAS summary statistics or can be estimated from a reference panel such as the 1000 Genomes Project [44]. Taken together, **Σ** can be estimated with

10 $$ {\hat{\Sigma}}_j=N{\left[\mathrm{SE}\left({\hat{\beta}}_j\right){\hat{\sigma}}_j^2\right]}^2 $$

11 $$ {\hat{\Sigma}}_{ji}={\hat{\beta}}_j{\hat{\beta}}_i{\hat{\sigma}}_j^2{\hat{\sigma}}_i^2 $$

where ${\hat{\sigma}}_j^2$ is an MAF-based estimator of $E\left({X}_j^2\right)$.

#### Step 1-c: Partition summary statistics for training and validation sets

After generating **x**^(*tr*)⊤^**y**^(*tr*)^ terms as described above from the conditional distribution, we can obtain the validating or testing summary statistics by

12 $$ {\mathbf{x}}^{(v)^{\mathrm{T}}}{\mathbf{y}}^{(v)}={\mathbf{x}}^{\mathrm{T}}\mathbf{y}\hbox{-} {\mathbf{x}}^{(tr)^{\mathrm{T}}}{\mathbf{y}}^{(tr)} $$

Consequently, subsampled GWAS summary statistics for the training set can be estimated by

13 $$ {{\hat{\beta}}_j}^{(tr)}={\left[\left(N-n\right){\hat{\sigma}}_j^2\right]}^{-1}{\mathbf{x}}^{(tr)^{\mathrm{T}}}{\mathbf{y}}^{(tr)} $$

14 $$ \mathrm{SE}\left({\hat{\beta_j}}^{(tr)}\right)=\sqrt{\frac{N}{N-n}}\mathrm{SE}\left(\hat{\beta_j}\right) $$

### Step 2: Evaluate model performance using GWAS summary data

#### Step 2-a: Calculate PRS prediction accuracy with summary statistics

Being able to generate summary statistics for the training and validation datasets resolves a critical issue in model tuning. However, challenges remain in evaluating PRS performance on the testing or validation set without individual-level data. Almost all the PRS approaches in the literature use a linear prediction model as follows:

15 $$ \hat{\mathbf{Y}}=\mathbf{Xw} $$

where **w**^⊤^ = (*w*_1_, …, *w*_*p*_) is the weight for SNPs in PRS. In a traditional PRS, marginal regression coefficients from GWAS are used as the weight values, i.e., $\mathbf{w}=\hat{\boldsymbol{\upbeta}}$, while in other PRS models the weight can be more sophisticated. Here, we demonstrate how to calculate *R*^2^, a commonly used metric to quantify PRS predictive performance, from subsampled GWAS summary data, but our method can be extended to other metrics (e.g., AUC [45]) as well. *R*^2^ on the validation dataset (**y**^(*v*)^, **x**^(*v*)^) can be calculated as

16 $$ {R}^2=\frac{{\left({\sum}_{i=1}^n{y}_i^{(v)}{\hat{y}}_i^{(v)}-n\overline{{\mathbf{y}}^{(v)}}\overline{{\hat{\mathbf{y}}}^{(v)}}\right)}^2}{\sum \limits_{i=1}^n{\left({y}_i^{(v)}-\overline{{\mathbf{y}}^{(v)}}\right)}^2{\sum}_{i=1}^n{\left({\hat{y}}_i^{(v)}-\overline{{\hat{\mathbf{y}}}^{(v)}}\right)}^2} $$

where ${\hat{\mathbf{y}}}^{(v)}={\mathbf{x}}^{(v)}\mathbf{w}\ \mathrm{and}\ \overline{{\hat{\mathbf{y}}}^{(v)}}$ is the sample mean of ${\hat{\mathbf{y}}}^{(v)}$. If the SNPs are pruned, it can be shown that the empirical variance of $\hat{\mathbf{Y}}$ can be approximated by

17 $$ \frac{1}{n}{\sum}_{i=1}^n{\left({\hat{y}}_i^{(v)}-\overline{{\hat{\mathbf{y}}}^{(v)}}\right)}^2\approx {\sum}_{j=1}^p{w}_j^2{\hat{\sigma}}_j^2 $$

Although empirical variance of *Y* does not affect model tuning, it affects the scale of *R*^2^ and is thus critical for interpreting the results. This term can be approximated by

18 $$ \frac{1}{n}{\sum}_{i=1}^n{\left({y}_i^{(v)}-\overline{{\mathbf{y}}^{(v)}}\right)}^2={\beta}_j^2E\left({X}_j^2\right)+E\left({\epsilon}_j^2\right),\forall j $$

Although Var(*Y*) is always greater than $E\left({\epsilon}_j^2\right)$ for any *j*, the gap between these two is negligible in real GWAS due to the small effect size of each individual SNP. Thus, a simple estimator for Var(*Y*) can be

19 $$ \frac{1}{n}{\sum}_{i=1}^n{\left({y}_i^{(v)}-\overline{{\mathbf{y}}^{(v)}}\right)}^2\approx {\max}_j\left[\frac{1}{N}{\hat{\varepsilon}}_j^T{\hat{\varepsilon}}_j\right]\approx N\kern0.28em {\max}_j\left[\mathrm{SE}{\left({\hat{\beta}}_j\right)}^2{\hat{\sigma}}_j^2\right] $$

Additionally, since we assumed data to be centered, the mean values in the numerator can be dropped. Taken together, *R*^2^ can be estimated as

20 $$ {R}^2\approx \frac{{\left(\frac{1}{n}{\sum}_{j=1}^p{w}_j{\mathbf{x}}_j^{(v)^{\mathrm{T}}}{\mathbf{y}}^{(v)}\right)}^2}{N\kern0.28em {\max}_j\kern0.28em \left[\mathrm{SE}{\left({\hat{\beta}}_j\right)}^2{\hat{\sigma}}_j^2\right]\kern0.28em {\sum}_{j=1}^p{w}_j^2{\hat{\sigma}}_j^2} $$

In practice, we use the 90% quantile of $\frac{{\hat{\varepsilon}}_j^{\top }{\hat{\varepsilon}}_j}{N-1}$, *j* = 1, 2, …*p*, as a more robust estimator for Var(*Y*).

#### Step 2-b: Model-tuning strategies

So far, we have introduced strategies to subsample association statistics on training and validation sets and evaluate model performance using GWAS summary statistics. Combining these two key steps, we will be able to perform model tuning using GWAS summary data. Suppose a PRS model uses GWAS marginal estimates $\hat{\boldsymbol{\upbeta}}$ as input and generates SNP weights ${w}_j\left(\hat{\boldsymbol{\upbeta}},\lambda \right)$ for each SNP. The goal is to find the optimal value of tuning parameter *λ* that maximizes the predictive accuracy. In the simple setting we introduced above, we will generate summary statistics for training and validation datasets. After specifying a tuning parameter *λ*, SNP weights in PRS can be trained by applying the model to the training summary statistics. Then, the prediction accuracy *R*^2^ on the validation summary statistics will be a function of *λ*. Therefore, we can select *λ* so that it maximizes model performance.

21 $$ \hat{\lambda}={\mathrm{argmax}}_{\lambda}\left({R}^2\left(\lambda \right)\right) $$

More generally, if the goal is to compare different models, both the summary statistics subsampling and performance evaluation steps remain unchanged. In this case, *R*^2^ will be a function of the model and we can choose the best-performing model by optimizing *R*^2^

22 $$ \hat{m}=\arg {\max}_{m=1,2\dots, M}\left({R}^2\left(\mathrm{model}\;\mathrm{m}\right)\right) $$

Furthermore, this framework can be used to conduct various types of model-tuning procedures. What we have laid out above is the simple training-validation data split approach. If one is interested in applying repeated learning, they can simply repeat the procedure (i.e., resampling training/validation datasets and evaluating *R*^2^ on the validation set) *K* times. The average *R*^2^ across *K* folds can be used to select the best model. Similarly, if *K*-fold cross-validation needs to be implemented, we can first independently simulate *K* − 1 sets of training subsample **x**^(*tr*, *k*)⊤^**y**^(*tr*, *k*)^ with sample size $\frac{N}{K}$. Then, we can obtain the *K*^*th*^ subsample by

23 $$ {\mathbf{x}}^{{\left(K, tr\right)}^T}{\mathbf{y}}^{\left(K, tr\right)}={\mathbf{x}}^{\mathrm{T}}\mathbf{y}\hbox{-} {\sum}_{k=1}^{K-1}{\mathbf{x}}^{{\left( tr,k\right)}^{\mathrm{T}}}{\mathbf{y}}^{\left( tr,k\right)} $$

Finally, rotate each one of the *K* subsamples as a validation sample and the rest as a training sample, and use the average *R*^2^ to select the best model. Taken together, PUMAS is a general framework that can perform a variety of model-tuning tasks.

### Simulation settings

We conducted simulations using real genotype data from WTCCC. The WTCCC dataset contains 15,918 samples with 393,273 genotyped SNPs across the whole genome. We removed SNPs that are not available in 1000 Genomes Project Phase III European samples from the simulations since 1000 Genomes data were used as the LD reference panel. We excluded individuals with genotype missingness rate higher than 0.01 and removed SNPs that satisfy any of the following conditions: (i) having minor allele frequency less than 0.01, (ii) having missing rate higher than 0.01 among all subjects, and (iii) having *p*-value from the Hardy-Weinberg equilibrium test lower than 1e−6. After quality control, 322,235 variants and 15,567 samples remained in the analyses. We first simulated effect sizes *β*_*j*_ from a normal distribution $N\left(0,\frac{h^2}{Mp}\right)$ where *h*^2^ is the heritability (fixed at 0.5), *M* is the total number of SNPs, and *p* is the proportion of causal variants. We chose two values of *p* (i.e., 0.001 and 0.1) to represent sparser and more polygenic genetic architecture. Following the LDAK paper [46], we then replaced the raw effect sizes by ${\beta}_j^{\ast }={\beta}_j{\left[2{p}_j\left(1-{p}_j\right)\right]}^{\alpha }$ where *α* =  − 2, − 1, 0, 1, 2 to better evaluate the performance of PUMAS under various genetic architecture. Thus, in total, we conducted simulations under 10 different settings. In each setting, causal SNPs were randomly selected across the genome and the effect sizes of non-causal SNPs were set to be 0. Using these simulated effect sizes, we generated continuous trait values in GCTA [47]. We then performed marginal linear regressions and obtained GWAS summary statistics using PLINK [48]. These summary statistics were used as input for PUMAS.

We compared PUMAS with repeated learning (i.e., Monte Carlo cross-validation). Instead of partitioning *N* samples into *k* non-overlapping folds, which is what a *k*-fold cross-validation does, repeated learning randomly selects $\frac{N\left(k-1\right)}{k}$ samples to form the training dataset and evaluates the model performance on the remaining $\frac{N}{k}$ samples. This procedure is then repeated *k* times to obtain an averaged prediction accuracy (i.e., *R*^2^) across *k* folds of analysis for each prediction model. Here, we implemented a 4-fold repeated learning approach. In each fold, we randomly select 75% of WTCCC samples (i.e., $\frac{3}{4}\times 15567\approx 11675$) to perform GWAS, and evaluate the predictive performance of PRS on the remaining 25% of individuals (i.e., 15567 − 11675 = 3892). We repeated this process 4 times and reported the average predictive *R*^2^ for each PRS model with different *p*-value cutoffs. For comparison, we implemented PUMAS in a similar fashion. Based on the GWAS summary data computed from all WTCCC samples, PUMAS generates a set of summary statistics for 75% of samples as the training data for PRS and evaluates the predictive performance of PRS on the corresponding validation summary statistics (i.e., summary statistics for the remaining 25% of samples). We repeated this procedure 4 times and reported the average *R*^2^ for each PRS model. In this simulation, we consider PRS models with *p*-value cutoffs ranging from 1e−5 to 1.

To show that PUMAS can be applied to binary traits, we conducted additional simulations under settings described above. For each simulation setting, we kept the same SNP effects, heritability, and proportion of causal variants. However, instead of generating quantitative phenotypes, we simulated binary phenotypes using a population prevalence of 50% and case-control ratio of 1:1 in GCTA [47]. We performed marginal logistic regressions in PLINK to obtain GWAS summary statistics. Like the simulation for quantitative traits, we compared the performance between PUMAS and 4-fold repeated learning using individual-level data for binary traits. We used the area under the ROC curve (AUC) as the metric to assess prediction accuracy in repeated learning.

Finally, to investigate the accuracy of our approximation for the covariance of **x**^*T*^**y**, we performed additional simulations in WTCCC. We implemented a total of 8 settings with different sample sizes, heritability, and proportion of causal variants. We simulated quantitative trait values and performed marginal linear regression to obtain GWAS summary statistics. Then, we calculated and compared diagonal elements Σ_*j*_ and ${\hat{\Sigma}}_j$, and off-diagonal elements Σ_*ji*_ and ${\hat{\Sigma}}_{ji}$, respectively. Note that the theoretical values of elements in the covariance matrix are shown in Eqs. (8–9), while the approximated values are shown in Eqs. (10–11). We assessed the approximation of both diagonal and off-diagonal elements. We found a very high correlation between ${\hat{\Sigma}}_j$ and Σ_*j*_ (greater than 0.99 in all of 8 simulation settings) and negligible off-diagonal elements (Additional file 1: Fig. S15; Additional file 13: Table. S12), which justifies our approximation procedure.

### GWAS data

GWAS summary statistics on EA was shared to us by Dr. Aysu Okbay. In this dataset, samples from Add Health, HRS, 23&me, and Wisconsin Longitudinal Study were excluded (*N* = 742,903; number of SNPs = 10,824,042). Imputed genotype data for Add Health (*N* = 9974; number of SNPs = 9,664,514) and HRS (*N* = 15,567; number of SNPs = 18,144,468) were accessed through dbGap (phs001367 and phs000428) and the EA phenotypes were defined following the SSGAC GWAS [19]. Both Add Health and HRS genotype data were imputed using 1000 Genome Project data as reference. The comprehensive data cleaning procedure is documented on the Add Health website at https://addhealth.cpc.unc.edu/wp-content/uploads/docs/user_guides/AH_GWAS_QC.pdf. For HRS, SNPs with imputation quality score < 0.8 were removed from the dataset. After matching samples with accessible phenotypic information, 4775 Add Health samples and 10,214 HRS samples with self-reported European ancestry were used to validate EA PRS. The IGAP 2013 AD GWAS (*N* = 54,162; number of SNPs = 7,055,881) dataset was accessed through the IGAP website (http://web.pasteur-lille.fr/en/recherche/u744/igap/igap_download.php). GWAS summary statistics (number of SNPs = 9,037,014) for 7050 ADGC samples can be accessed through the NIAGADS database (NG00076). Predictive performance on ADGC samples was assessed using summary statistics-based *R*^2^. Following a recent paper, we constructed the AD-proxy phenotype in the UK Biobank based on each sample’s AD status, AD history of parents, whether parents are still alive, and parental age (or age at death) [24]. Imputed genotype data (*N* = 355,583; number of SNPs = 9,605,099) were accessed through the UKB. The UKB genotype data was imputed to the Haplotype Reference Consortium reference. We removed samples who are not of European ancestry and SNPs with minor allele frequency < 0.01 or imputation *R*^2^ < 0.9. In addition, we applied PUMAS to benchmark PRS performance on 65 GWASs. Details on these studies are summarized in Additional files 7, 8, and 9: Table. S6-S8.

For all PUMAS analysis throughout the paper, we first extracted SNPs intersected with the 1000 Genome Phase III data of European ancestry [44]. Then, we pruned GWAS summary statistics by a LD-block window size of 100 variants, a step size of 5 variants to shift windows, and a pairwise LD (i.e., *r*^2^) threshold of 0.1 using PLINK [48]. We used samples of European ancestry in the 1000 Genome Project Phase III as the reference panel to estimate LD. For GWASs that do not report MAF in the summary statistics, we estimated MAF from 1000 Genome project European samples. In addition, for the analysis of EA and AD, we also intersected GWAS summary statistics with SNPs in the validation set before LD-pruning. A *p*-value grid was used to search for the optimal *p*-value cutoff (Additional file 4: Table. S3).

### Identifying neuroimaging traits associated with AD

GWAS results for imaging traits were accessed from https://med.sites.unc.edu/bigs2/data/. The IGAP 2019 AD GWAS summary statistics was accessed via NIAGADS (NG00075). We constructed the AD-proxy phenotype in the UK Biobank following a recent paper [24]. To avoid sample overlap between GWASs, we inferred individuals in the UK Biobank who have undergone brain MRI scans and removed them from the AD-proxy GWAS. All individuals who have visited at least one of the UKB imaging centers were removed from the analysis. 318,773 independent samples remained after removing imaging samples from the data. We performed GWAS with the first 12 principal components [49], age, sex, genotyping array, and assessment center as covariates. To test if our approach to remove overlapping samples between neuroimaging GWAS and the AD-proxy analysis was effective, we used cross-trait LD score regression to estimate the intercepts between 211 imaging traits and the AD-proxy GWAS (Additional file 1: Fig. S13) [50]. BADGERS software was used to conduct the imaging trait PRS-AD association analysis [30]. Meta-analysis was conducted using the sample size-weighted approach [51].

## Supplementary Information

**Additional file 1:** Supplementary notes and figures.

**Additional file 2: Table S1.** Compare model tuning techniques in WTCCC simulation with continuous phenotype.

**Additional file 3: Table S2.** Compare model tuning techniques in WTCCC simulation with binary phenotype.

**Additional file 4: Table S3.** R^2^ based on PUMAS and external validations for EA and AD.

**Additional file 5: Table S4.** PUMAS results on LDL cholesterol and EA with various sample size specifications.

**Additional file 6: Table S5.** Predictive performance on external validation for AD PRS based on pruned and clumped summary statistics.

**Additional file 7: Table S6.** Summary of optimized 65 GWAS summary statistics.

**Additional file 8: Table S7.** Summary of improvement of optimized 65 GWAS summary statistics comparing to PRS with arbitrary *p*-value cutoff.

**Additional file 9: Table S8.** Computation time for analysis of 65 publicly available GWAS summary statistics.

**Additional file 10: Table S9.** Summary of fine-tuned PRSs for UK Biobank imaging traits.

**Additional file 11: Table S10.** Cross-trait LD score regression intercept estimates for 211 imaging traits with UKBB AD-proxy GWAS.

**Additional file 12: Table S11.** Complete association results between 211 neuroimaging trait PRSs and AD.

**Additional file 13: Table S12.** Simulation settings for investigating the estimation accuracy of covariance of summary statistics.

**Additional file 14.** Review history.

## Data Availability

The Add Health study data and HRS study data were accessed through dbGap (http://dbgap.ncbi.nlm.nih.gov) with accession codes phs001367 and phs000428 [20, 21]. The UKB data were downloaded from UK Biobank Resource (https://www.ukbiobank.ac.uk) under application number 42148 [52]. The PUMAS software is available at https://github.com/qlu-lab/PUMAS [53]. The PUMAS source code used in this study is deposited at https://zenodo.org/record/5202800#.YRmC3y2cbRZ with DOI: 10.5281/zenodo.5202800. The PUMAS package and source code are under MIT license. This study used existing software and tools for data analysis. LDSC can be downloaded from https://github.com/bulik/ldsc [54]. GCTA can be downloaded from https://cnsgenomics.com/software/gcta/#Download [47]. PLINK version 1.9 can be downloaded from https://www.cog-genomics.org/plink/1.9/ [48]. PRSice-2 can be downloaded from https://www.prsice.info [55].
