# Supplementary material for: PUMAS: fine-tuning polygenic risk scores with GWAS summary statistics
Source: Genome Biol. 2021 Sep 6;22:257. doi: 10.1186/s13059-021-02479-9 (PMC8419981; doi:10.1186/s13059-021-02479-9)
Supplement: Supplementary file 1 — Additional file 1: Supplementary notes and figures. [file 13059_2021_2479_MOESM1_ESM.docx]

**Supplementary Notes**

**The derivation of distribution of** $\mathbf{x}^{(tr)^{T}}\mathbf{y}^{\left( tr \right)}|\mathbf{x}^{T}\mathbf{y}$

We derive the distribution of $\mathbf{x}^{(tr)^{T}}\mathbf{y}^{\left( tr \right)}|\mathbf{x}^{T}\mathbf{y}$ using the formula for conditional distribution of two multivariate normal random vectors. For example, if $\mathbf{X}\sim N\left( \boldsymbol{\mu}_{\mathbf{X}},\boldsymbol{\Sigma}_{\mathbf{X}} \right)$ and $\mathbf{Y}\sim N\left( \boldsymbol{\mu}_{\mathbf{Y}}, \boldsymbol{\Sigma}_{\mathbf{Y}} \right)$, and $cov\left( \mathbf{X},\mathbf{Y} \right)=\boldsymbol{\Sigma}_{\mathbf{XY}}$, then the distribution of $\mathbf{X}|\mathbf{Y}$ also follows a multivariate normal distribution with mean and covariance matrix

$$E\left( \mathbf{X} | \mathbf{Y}=\mathbf{y} \right)=\boldsymbol{\mu}_{\mathbf{X}}+\boldsymbol{\Sigma}_{\mathbf{XY}}\boldsymbol{\Sigma}_{\mathbf{Y}}^{-1}\left( \mathbf{y}-\boldsymbol{\mu}_{\mathbf{Y}} \right)$$

$$Var\left( \mathbf{X} | \mathbf{Y}=\mathbf{y} \right)=\boldsymbol{\Sigma}_{\mathbf{X}}-\boldsymbol{\Sigma}_{\mathbf{XY}} \boldsymbol{\Sigma}_{\mathbf{Y}}^{-1} \boldsymbol{\Sigma}_{\mathbf{YX}}$$

Based on this property, since

$$\mathbf{x}^{T}\mathbf{y}\sim N(NE(\mathbf{X}^{T}Y),NVar(\mathbf{X}^{T}Y))$$

$$\mathbf{x}^{(tr)^{T}}\mathbf{y}^{(tr)}\sim N((N-n)E(\mathbf{X}^{T}Y),(N-n)Var(\mathbf{X}^{T}Y))$$

and the covariance between $\mathbf{x}^{(tr)^{T}}\mathbf{y}^{(tr)}$ and $\mathbf{x}^{T}\mathbf{y}$ is

$$\begin{matrix} cov(\mathbf{x}^{(tr)^{T}}\mathbf{y}^{(tr)},\mathbf{x}^{T}\mathbf{y}) & =cov(\mathbf{x}^{(tr)^{T}}\mathbf{y}^{(tr)},\mathbf{x}^{(tr)^{T}}\mathbf{y}^{(tr)}+\mathbf{x}^{(v)^{T}}\mathbf{y}^{(v)}) \\ & =Var(\mathbf{x}^{(tr)^{T}}\mathbf{y}^{(tr)}) \\ & =(N-n)Var(\mathbf{X}^{T}Y) \end{matrix}$$

Then we can write the expectation of the distribution of $\mathbf{x}^{(tr)^{T}}\mathbf{y}^{\left( tr \right)}|\mathbf{x}^{T}\mathbf{y}$ as

$$E(\mathbf{x}^{(tr)^{T}}\mathbf{y}^{(tr)}|\mathbf{x}^{T}\mathbf{y})=(N-n)E(\mathbf{X}^{T}Y)+\frac{N-n}{N}Var(\mathbf{X}^{T}Y)Var(\mathbf{X}^{T}Y)^{-1}(\mathbf{x}^{T}\mathbf{y}-NE(\mathbf{X}^{T}Y))$$

Replace $NE(\mathbf{X}^{T}Y)$ by the observed vector $\mathbf{x}^{T}\mathbf{y}$, we get

$$E(\mathbf{x}^{(tr)^{T}}\mathbf{y}^{(tr)}|\mathbf{x}^{T}\mathbf{y})=\frac{N-n}{N}\mathbf{x}^{T}\mathbf{y}$$

And the variance of the conditional distribution is

$$\begin{matrix} Var(\mathbf{x}^{(tr)^{T}}\mathbf{y}^{(tr)}|\mathbf{x}^{T}\mathbf{y}) & =(N-n)Var(\mathbf{X}^{T}Y)-\frac{N-n}{N}Var(\mathbf{X}^{T}Y)Var(\mathbf{X}^{T}Y)^{-1}(N-n)Var(\mathbf{X}^{T}Y) \\ & =\frac{(N-n)n}{N}Var(\mathbf{X}^{T}Y) \end{matrix}$$

Replace $Var\left( \mathbf{X}^{T}Y \right)$ by the observed covariance matrix $\boldsymbol{\Sigma}$, then

$$Var\left( \mathbf{x}^{(tr)^{T}}\mathbf{y}^{\left( tr \right)} | \mathbf{x}^{T}\mathbf{y} \right)=\frac{\left( N-n \right)n}{N} \boldsymbol{\Sigma}$$

**Supplementary Figures**

**
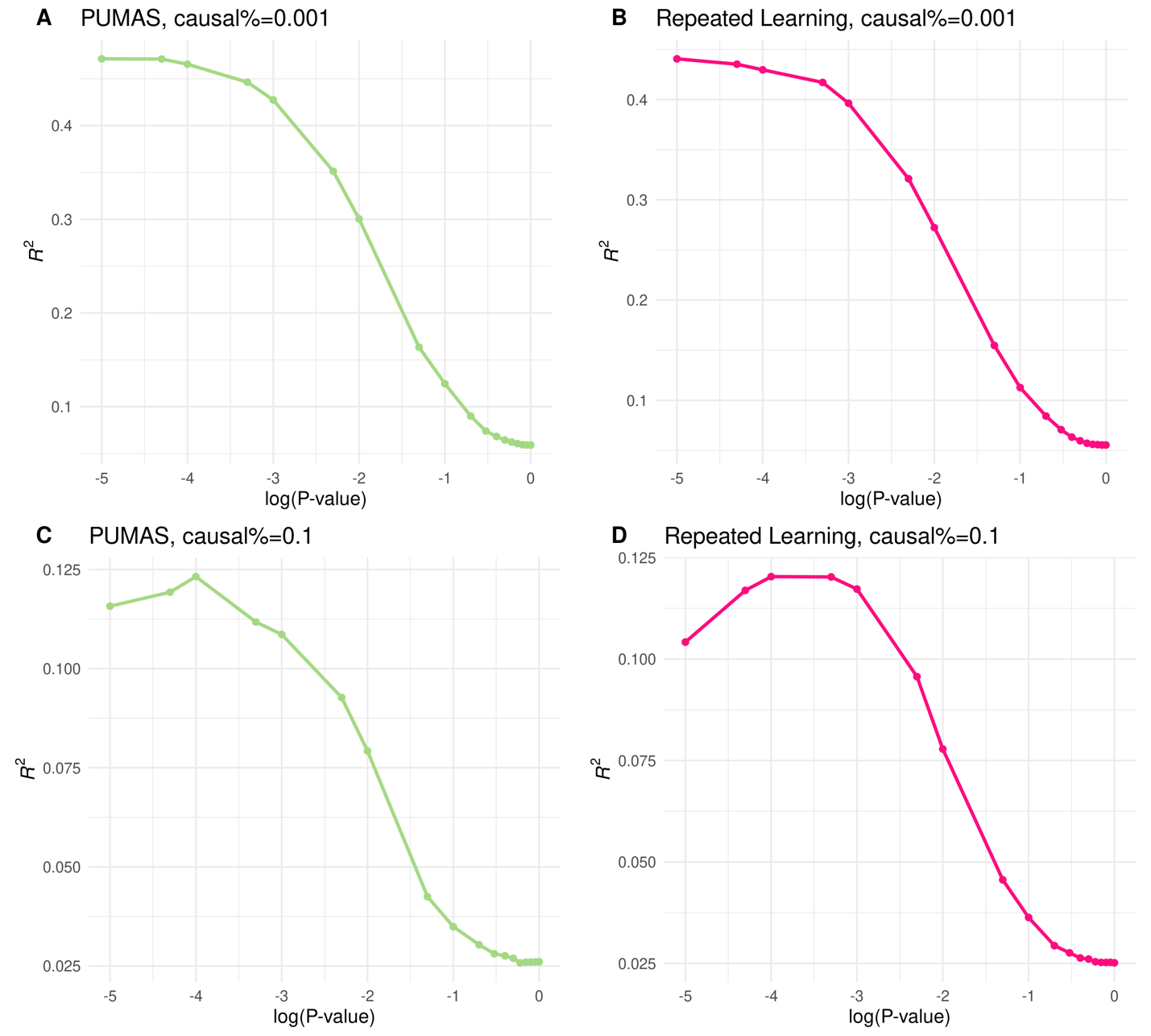
**

**Fig S1: Comparison of two model-tuning strategies in WTCCC samples under alpha = -2. (A)** PUMAS performance under a causal variant proportion of 0.001. **(B)** Repeated learning approach with individual-level data as input under a causal variant proportion of 0.001. **(C)** PUMAS performance under a causal variant proportion of 0.1. **(D)** Repeated learning approach with individual-level data as input under a causal variant proportion of 0.1. The X-axis shows the log-transformed p-value thresholds. The Y-axis shows the predictive performance quantified by average $R^{2}$ across four folds.**
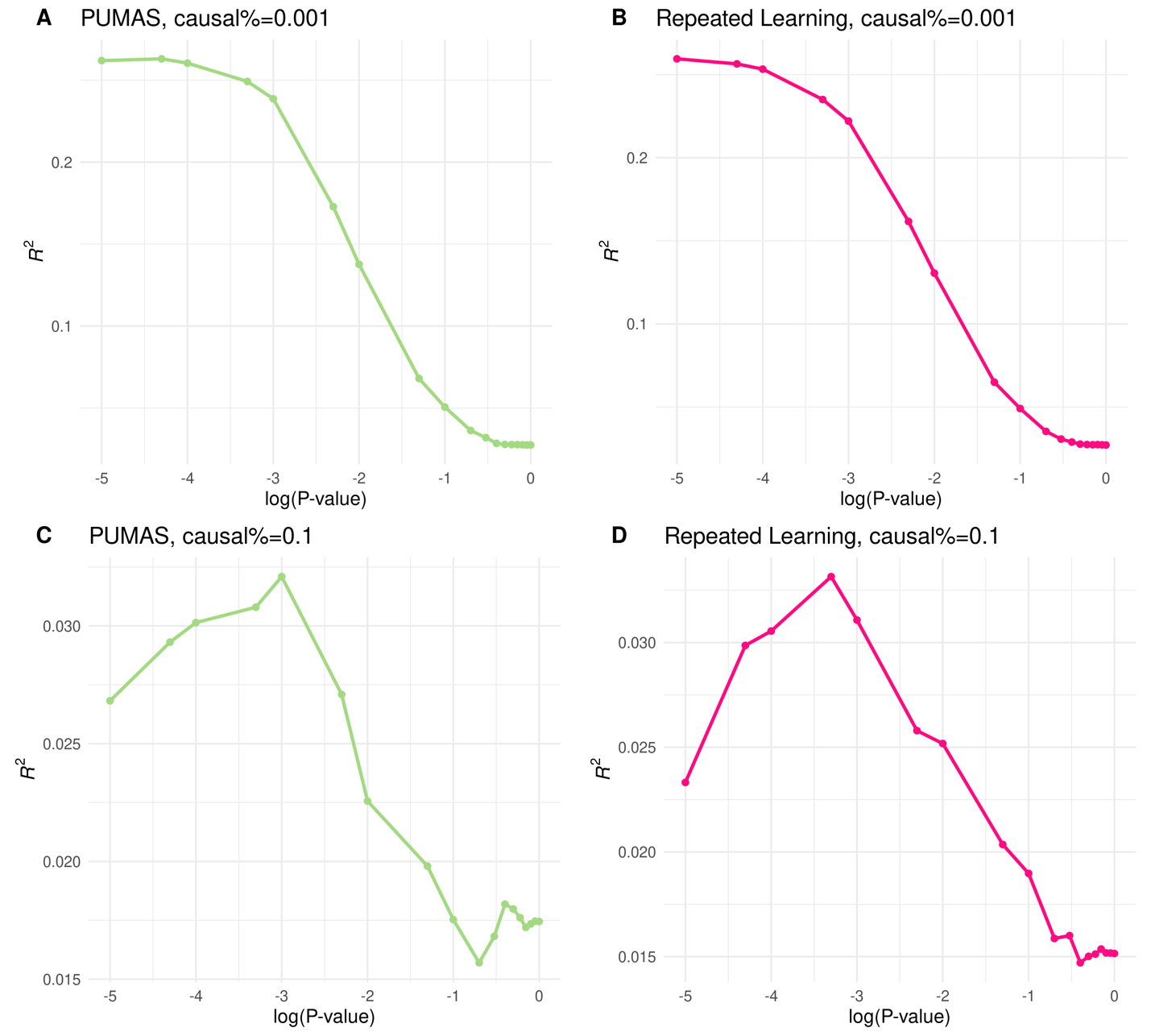
**

**Fig S2: Comparison of two model-tuning strategies in WTCCC samples under alpha = -1. (A)** PUMAS performance under a causal variant proportion of 0.001. **(B)** Repeated learning approach with individual-level data as input under a causal variant proportion of 0.001. **(C)** PUMAS performance under a causal variant proportion of 0.1. **(D)** Repeated learning approach with individual-level data as input under a causal variant proportion of 0.1. The X-axis shows the log-transformed p-value thresholds. The Y-axis shows the predictive performance quantified by average $R^{2}$ across four folds.

**
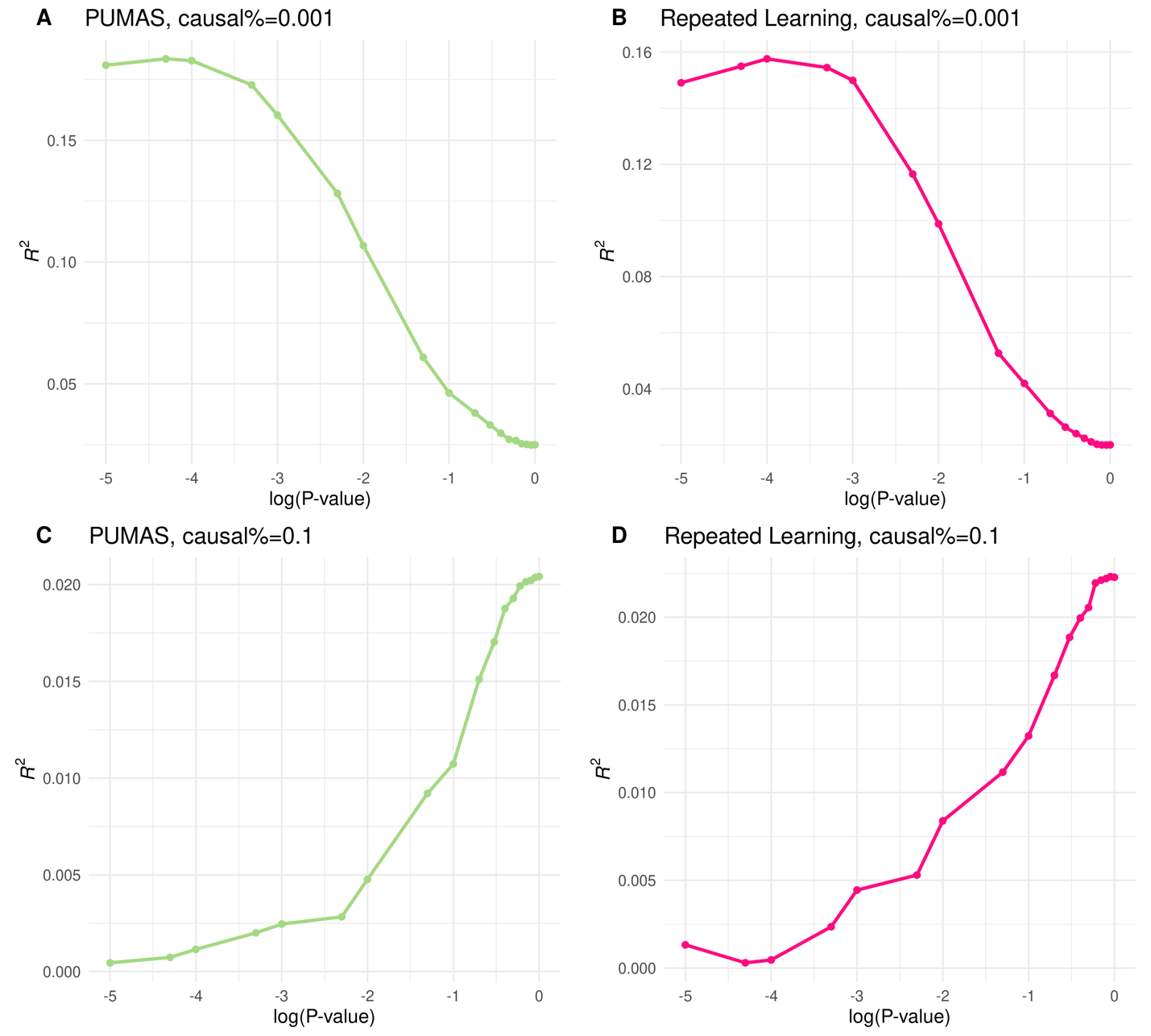
**

**Fig S3: Comparison of two model-tuning strategies in WTCCC samples under alpha = 1. (A)** PUMAS performance under a causal variant proportion of 0.001. **(B)** Repeated learning approach with individual-level data as input under a causal variant proportion of 0.001. **(C)** PUMAS performance under a causal variant proportion of 0.1. **(D)** Repeated learning approach with individual-level data as input under a causal variant proportion of 0.1. The X-axis shows the log-transformed p-value thresholds. The Y-axis shows the predictive performance quantified by average $R^{2}$ across four folds.

**
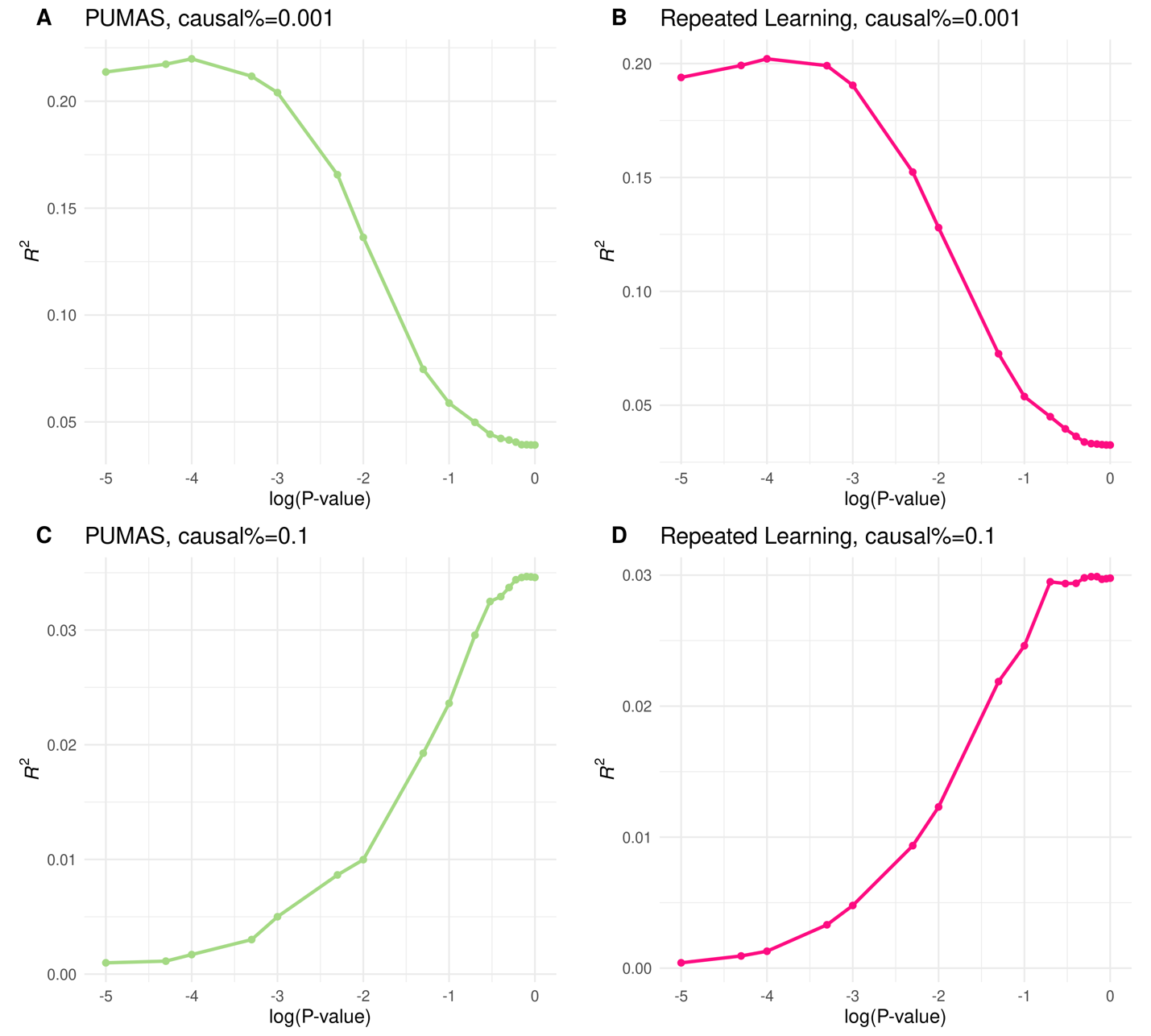
**

**Fig S4: Comparison of two model-tuning strategies in WTCCC samples under alpha = 2. (A)** PUMAS performance under a causal variant proportion of 0.001. **(B)** Repeated learning approach with individual-level data as input under a causal variant proportion of 0.001. **(C)** PUMAS performance under a causal variant proportion of 0.1. **(D)** Repeated learning approach with individual-level data as input under a causal variant proportion of 0.1. The X-axis shows the log-transformed p-value thresholds. The Y-axis shows the predictive performance quantified by average $R^{2}$ across four folds.

**
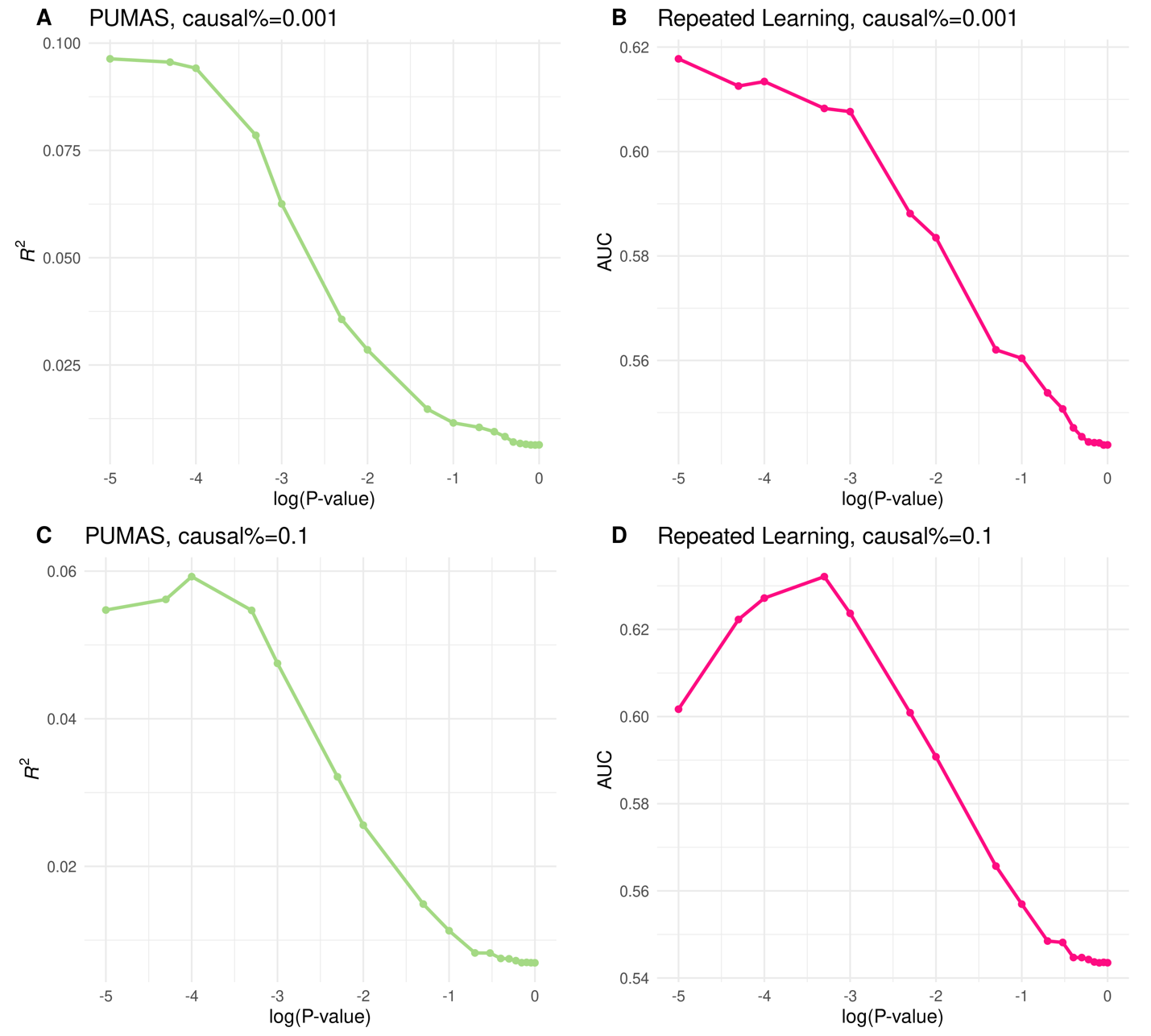
**

**Fig S5: Comparison of two model-tuning strategies for binary traits in WTCCC samples under alpha = -2. (A)** PUMAS performance under a causal variant proportion of 0.001. **(B)** Repeated learning approach with individual-level data as input under a causal variant proportion of 0.001. **(C)** PUMAS performance under a causal variant proportion of 0.1. **(D)** Repeated learning approach with individual-level data as input under a causal variant proportion of 0.1. The X-axis shows the log-transformed p-value thresholds. The Y-axis shows the predictive performance quantified by average $R^{2}$ for PUMAS and AUC for repeated learning across four folds.

**
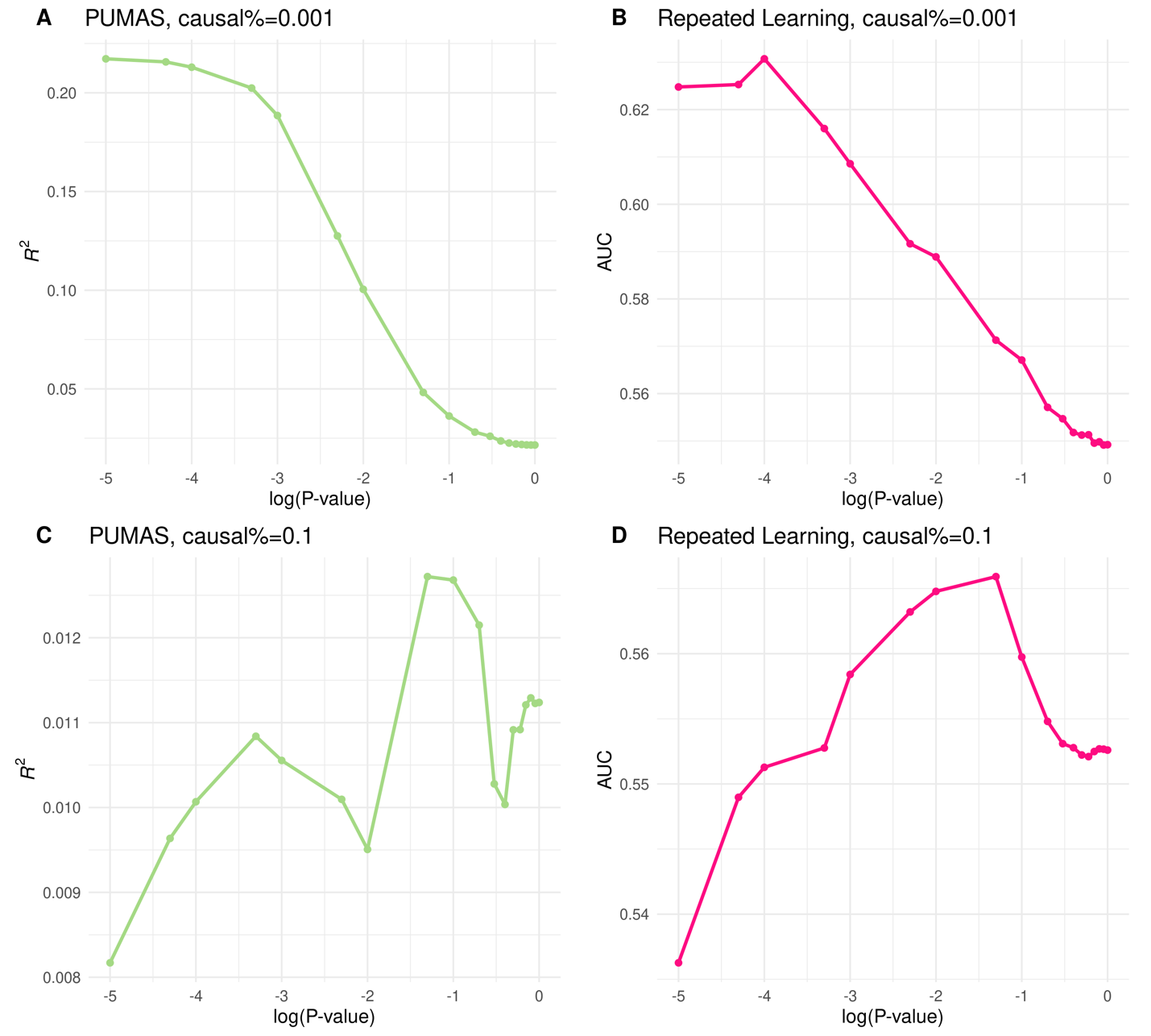
**

**Fig S6: Comparison of two model-tuning strategies for binary traits in WTCCC samples under alpha = -1. (A)** PUMAS performance under a causal variant proportion of 0.001. **(B)** Repeated learning approach with individual-level data as input under a causal variant proportion of 0.001. **(C)** PUMAS performance under a causal variant proportion of 0.1. **(D)** Repeated learning approach with individual-level data as input under a causal variant proportion of 0.1. The X-axis shows the log-transformed p-value thresholds. The Y-axis shows the predictive performance quantified by average $R^{2}$ for PUMAS and AUC for repeated learning across four folds.

**
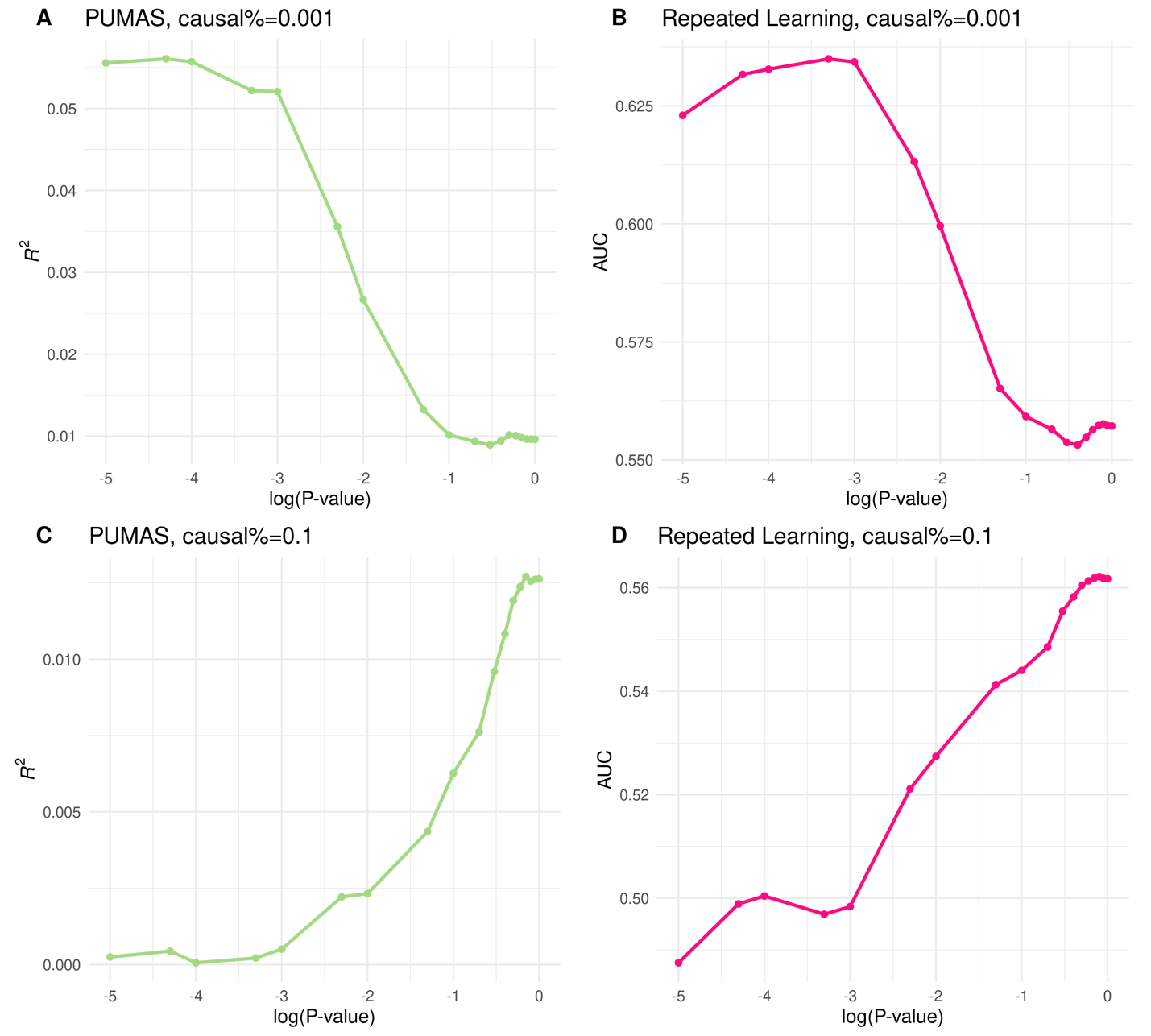
**

**Fig S7: Comparison of two model-tuning strategies for binary traits in WTCCC samples under alpha = 0. (A)** PUMAS performance under a causal variant proportion of 0.001. **(B)** Repeated learning approach with individual-level data as input under a causal variant proportion of 0.001. **(C)** PUMAS performance under a causal variant proportion of 0.1. **(D)** Repeated learning approach with individual-level data as input under a causal variant proportion of 0.1. The X-axis shows the log-transformed p-value thresholds. The Y-axis shows the predictive performance quantified by average $R^{2}$ for PUMAS and AUC for repeated learning across four folds.

**
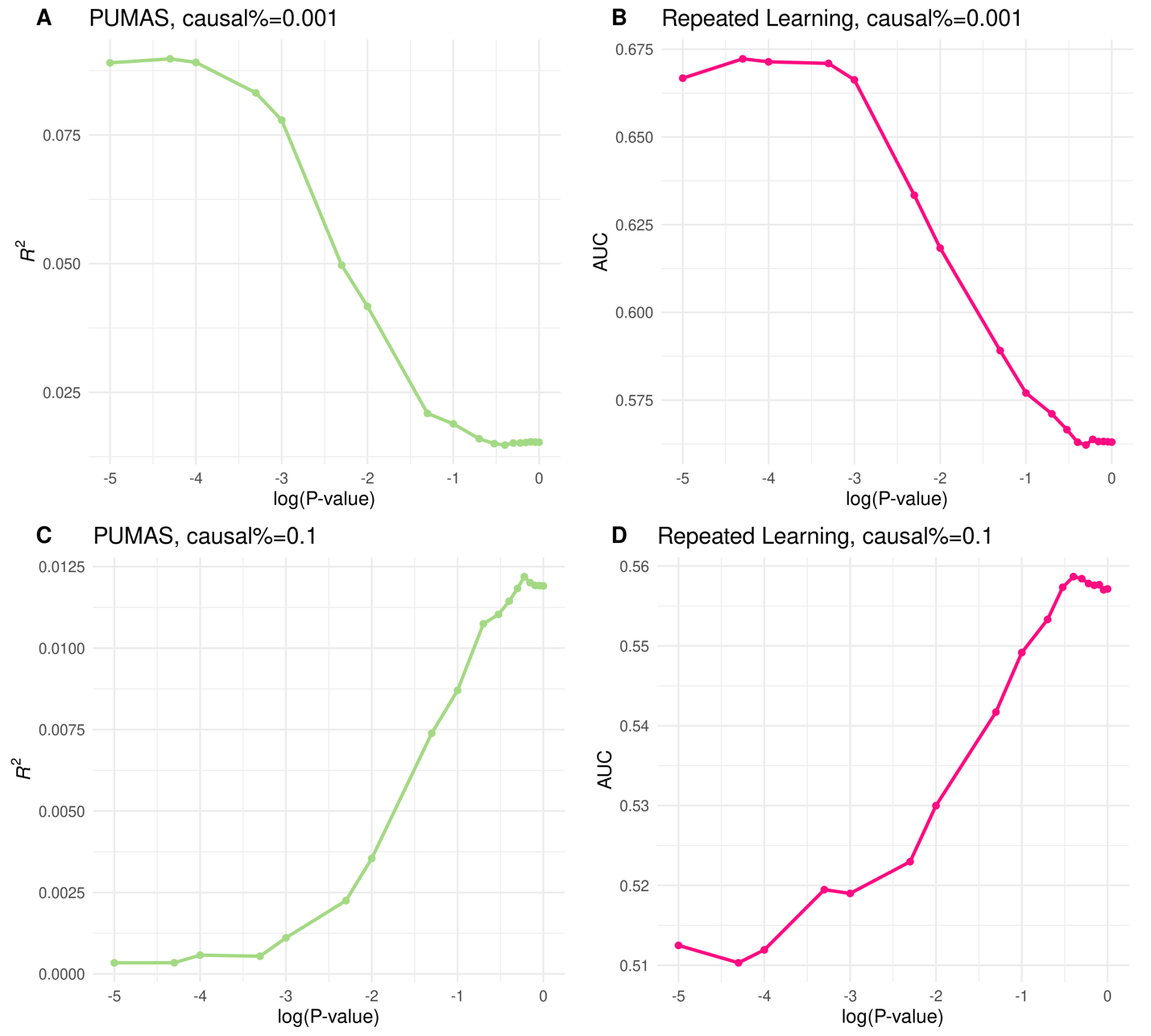
**

**Fig S8: Comparison of two model-tuning strategies for binary traits in WTCCC samples under alpha = 1. (A)** PUMAS performance under a causal variant proportion of 0.001. **(B)** Repeated learning approach with individual-level data as input under a causal variant proportion of 0.001. **(C)** PUMAS performance under a causal variant proportion of 0.1. **(D)** Repeated learning approach with individual-level data as input under a causal variant proportion of 0.1. The X-axis shows the log-transformed p-value thresholds. The Y-axis shows the predictive performance quantified by average $R^{2}$ for PUMAS and AUC for repeated learning across four folds.**
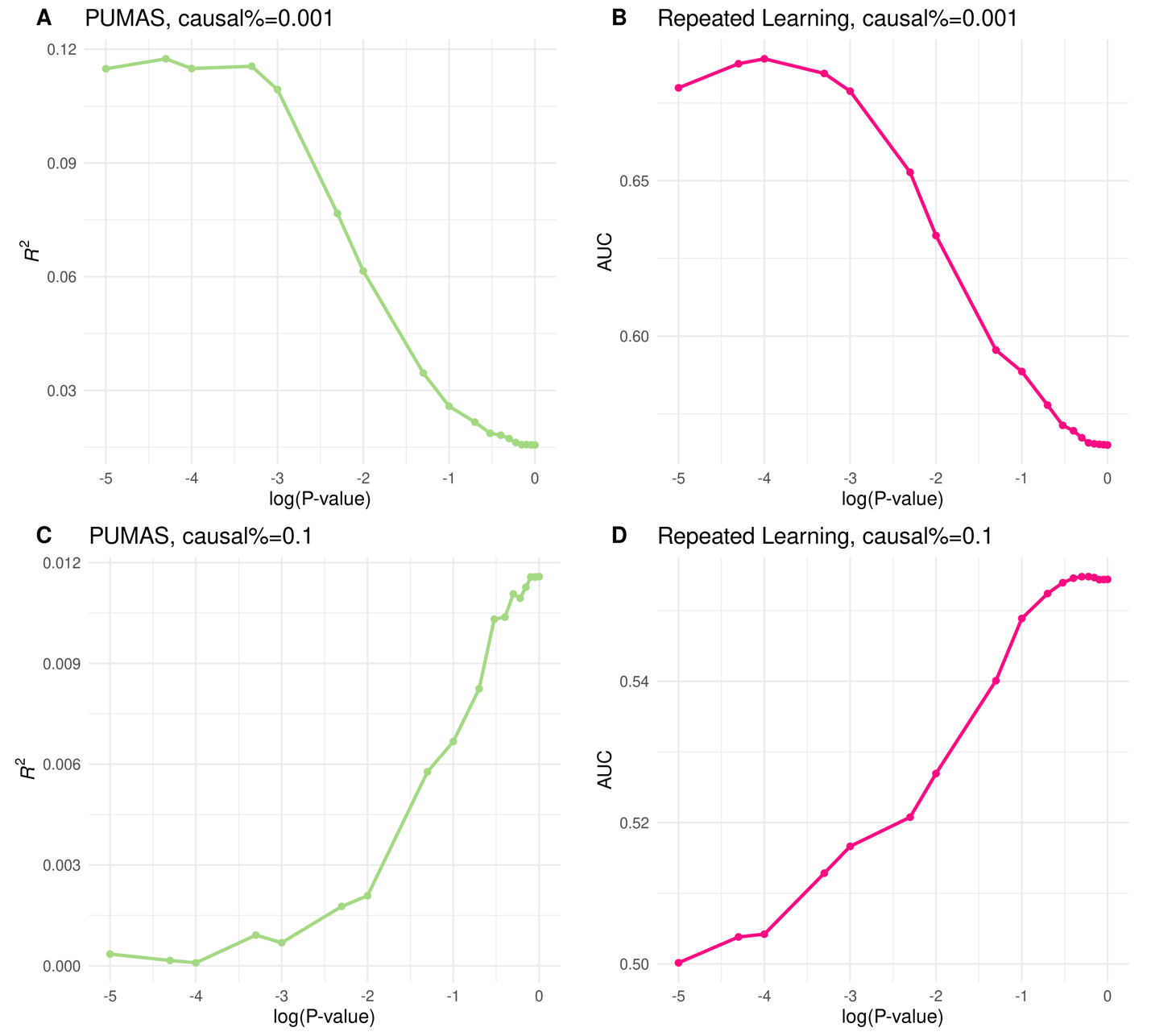
**

**Fig S9: Comparison of two model-tuning strategies for binary traits in WTCCC samples under alpha = 2. (A)** PUMAS performance under a causal variant proportion of 0.001. **(B)** Repeated learning approach with individual-level data as input under a causal variant proportion of 0.001. **(C)** PUMAS performance under a causal variant proportion of 0.1. **(D)** Repeated learning approach with individual-level data as input under a causal variant proportion of 0.1. The X-axis shows the log-transformed p-value thresholds. The Y-axis shows the predictive performance quantified by average $R^{2}$ for PUMAS and AUC for repeated learning across four folds.


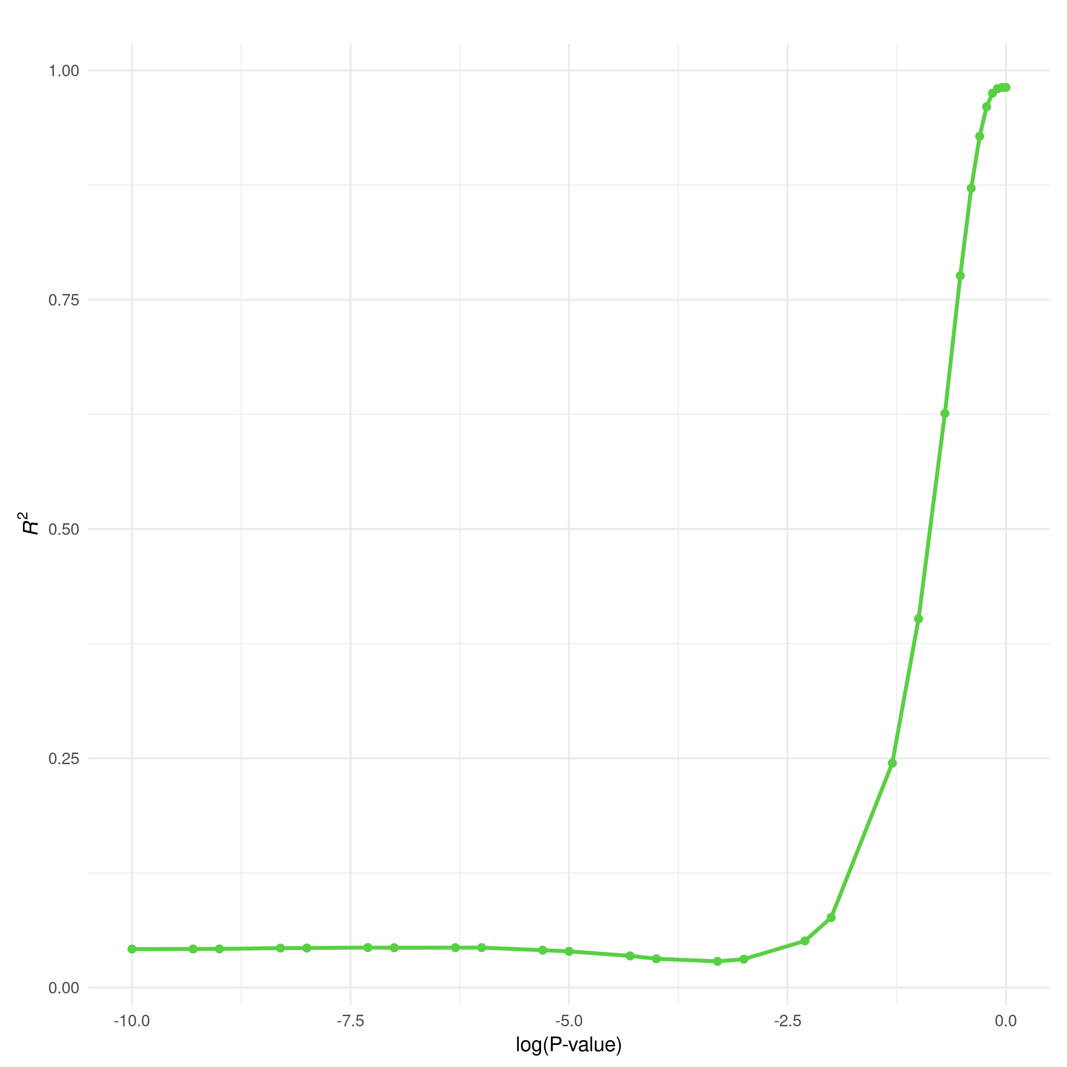


**Fig S10: PUMAS result using clumped IGAP 2013 AD GWAS as input.**


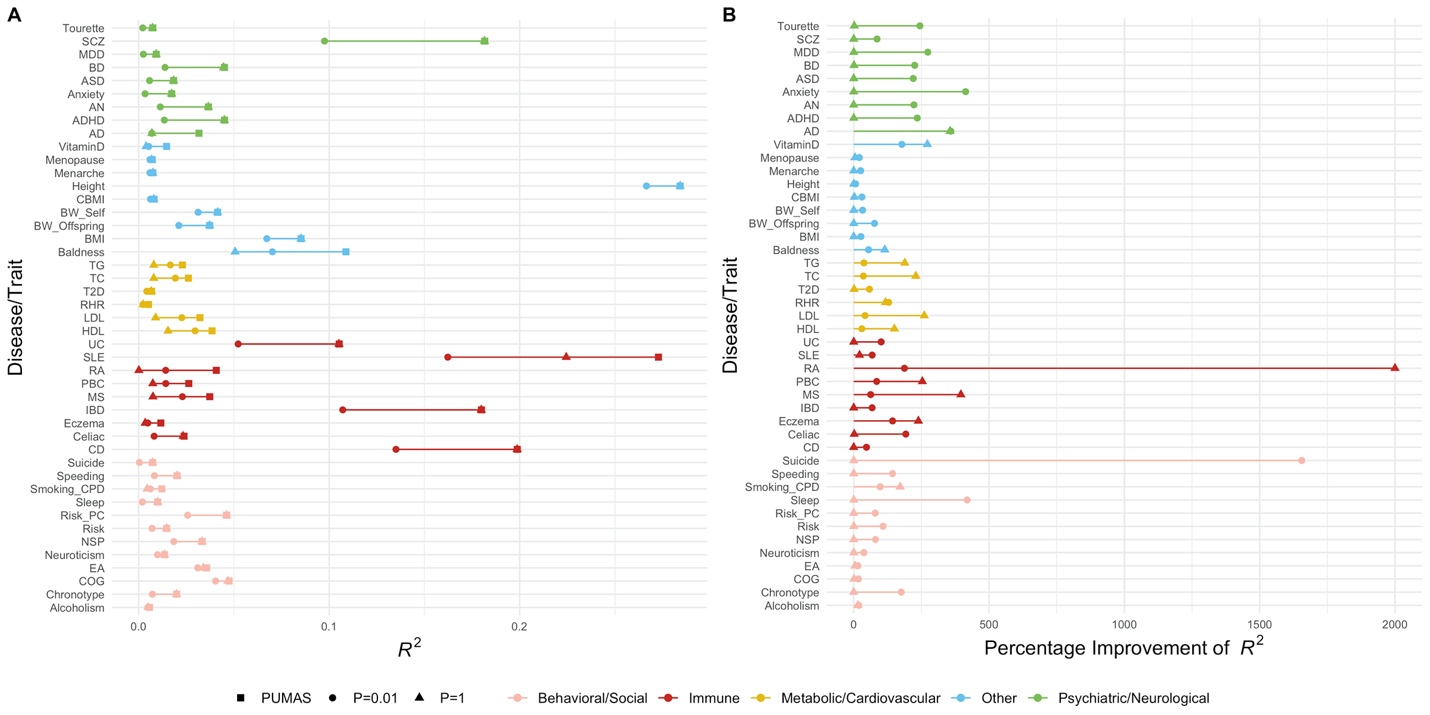
 **Fig S11: Improvement of predictive R^2^ of optimized 45 traits. (A)** PUMAS’s increase in predictive *R^2^* comparing to PRS of P=0.01 and P=1 **(B)** PUMAS’s percentage improvement in predictive *R^2^* comparing to PRS of P=0.01 and P=1. The percentage improvement of RA’s predictive performance by PUMAS comparing to its PRS at P=0.01 is truncated to be 2000% in panel B.


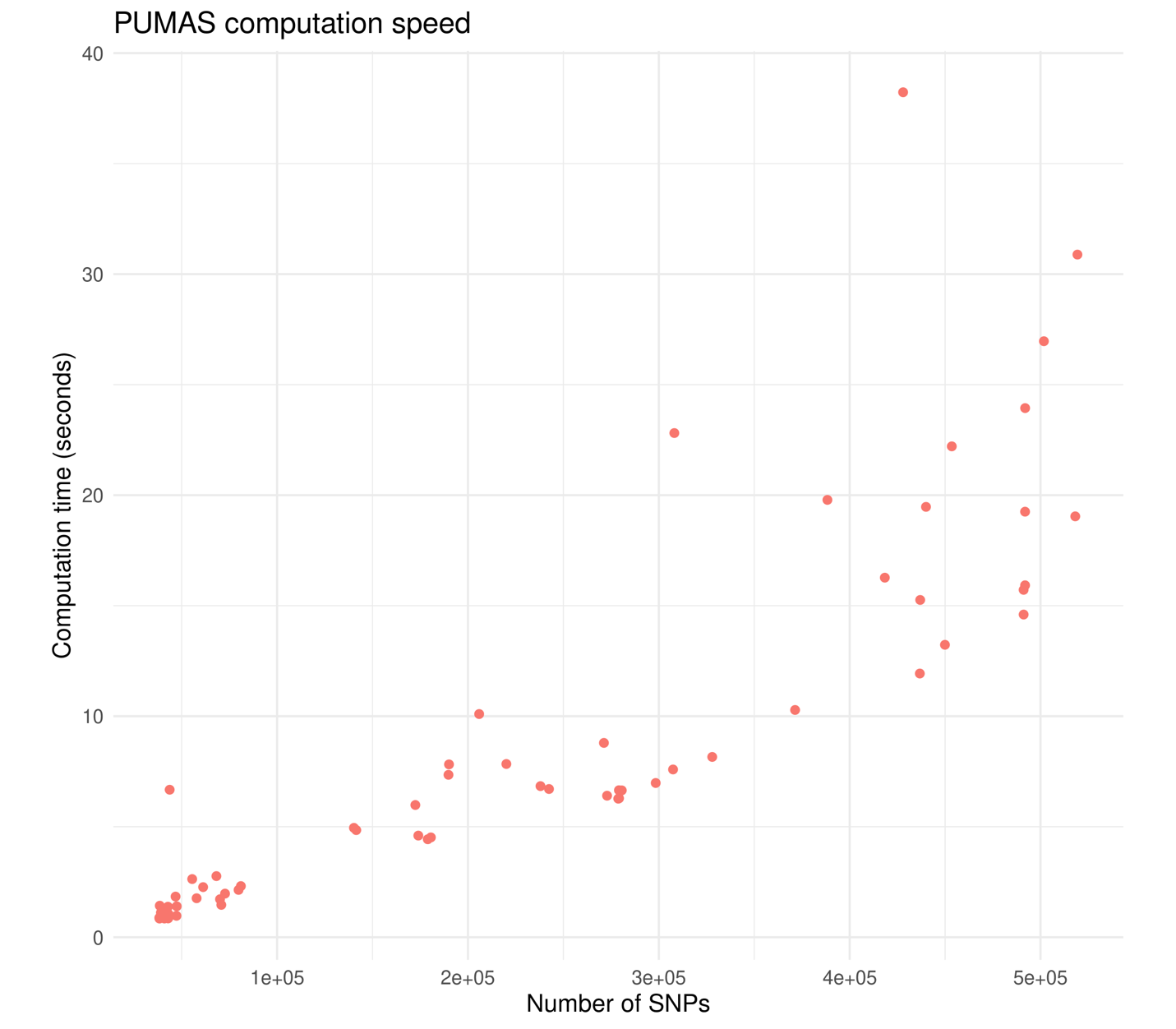


**Fig S12: Computation time for the analysis of 65 GWAS traits.** The X-axis shows the number of SNPs in the pruned GWAS. The Y-axis shows the elapsed computation time in seconds.


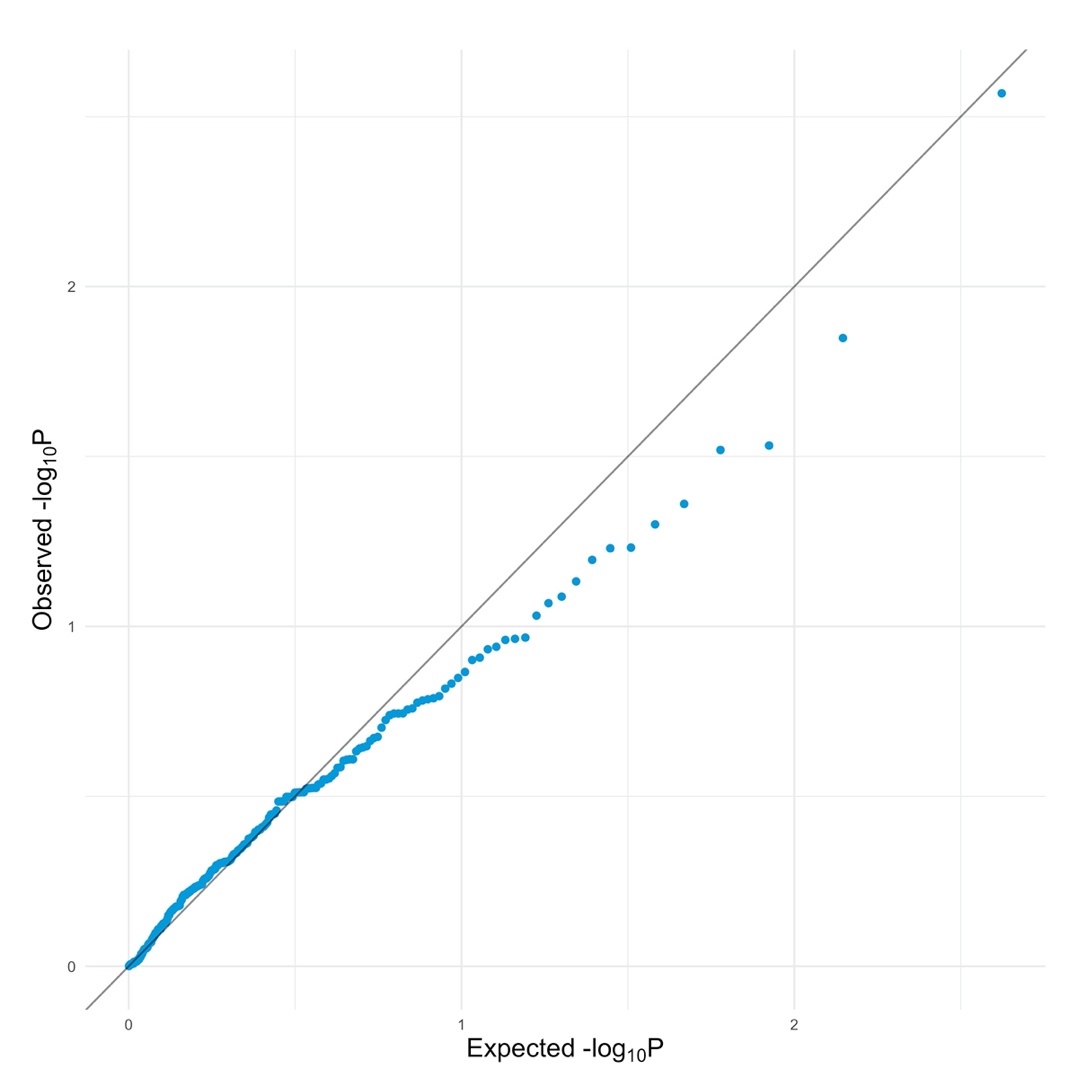


**Fig S13: QQ plot for p-values of LDSC intercept estimates between non-imaging AD-proxy GWAS and UK Biobank imaging traits.** P-value for the one-sample t-test of null hypothesis that the mean of LDSC intercepts equals zero is 0.3191.


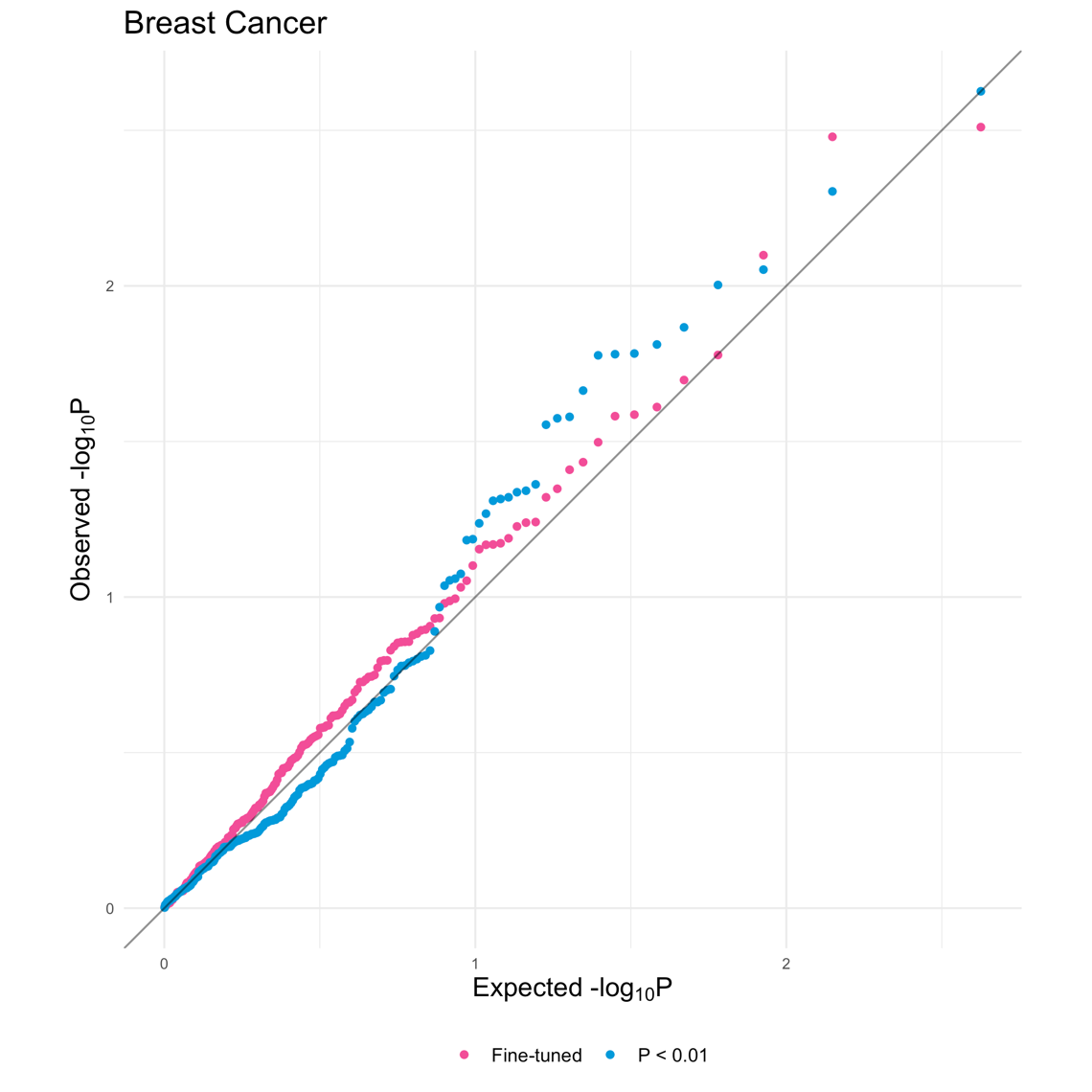


**Fig S14: QQ plot for associations between breast cancer and UK Biobank neuroimaging traits.**


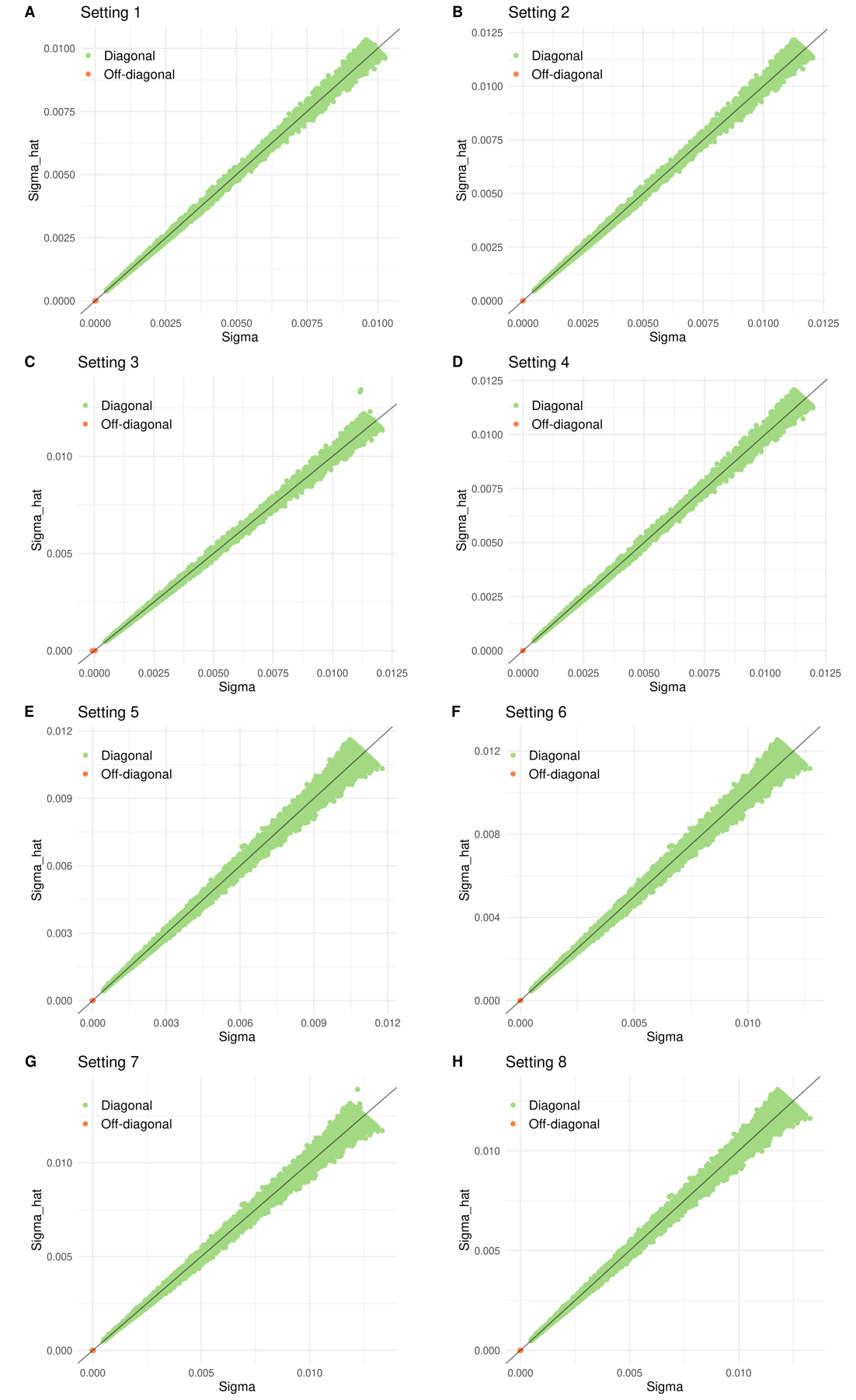


**Fig S15: Comparison of PUMAS’s approximated** $\boldsymbol{\Sigma}$ **and theoretical** $\boldsymbol{\Sigma}$ **in 8 simulation settings.** (**A-H**) scatter plots of approximated diagonal and off-diagonal elements versus theoretical diagonal and off-diagonal elements in each setting. Details on simulations settings are discussed in the **Methods** section.
