# Supplementary material for: PUMAS: fine-tuning polygenic risk scores with GWAS summary statistics
Source: Genome Biol. 2021 Sep 6;22:257. doi: 10.1186/s13059-021-02479-9 (PMC8419981; doi:10.1186/s13059-021-02479-9)
Supplement: Supplementary file 14 — Additional file 14. Review history. [file 13059_2021_2479_MOESM14_ESM.docx]

Review History

**First round of review**

**Reviewer 1**

**Were you able to assess all statistics in the manuscript, including the appropriateness of statistical tests used?** No

**Were you able to directly test the methods?** Yes

**Comments to author:**

Fine-tuning Polygenic Risk Scores with GWAS Summary Statistics

Zhao et al.

This is an interesting study proposing a novel method (PUMAS) to select an association p-value threshold for genome-wide SNPs that provides a better accuracy of polygenic risk prediction. To select an optimised p-value threshold, PUMA can obtain R^2 in a validation set, based on the conditional distribution of estimated SNP effects, which are derived from GWAS summary stats, without accessing the individual genotypes of the training dataset. The theoretical R^2 are evaluated to select an optimised p-value threshold that may result in the highest prediction accuracy.

This is a neat and nice theoretical work. However, I have a lot of problems in reading the manuscript particularly for the derivation and description of the method (pages 19 - 22). The authors would have provided an evidence that their derived theory is valid, and approximations used in their derivation are robust, which is missing in the current manuscript. Discussion should be improved in general. I have a number of questions and comments.

1. Method overview. Figure 1B can be improved, e.g. the authors can indicate the process includes to derive the conditional distribution of estimated SNP effects, given the GWAS summary stats, and simulate GWAS summary stats for any number of training and validation datasets. In the text (page 5), please add equation numbers (shown in Methods) for the first and second steps so that readers can easily understand what the steps are.

2. In simulations, I would suggest varying the genetic architecture of trait, e.g. the causal effects are proportional to the minor allele frequencies (SNP effect* = SNP effect * (2p(1-p))^alpha where p is the reference allele frequency and alpha is the scale parameter to determine the genetic architecture. Please see equation 5 and Figure 2 in the following paper.

Improved Heritability Estimation from Genome-wide SNPs. AJHG: 91: 1011.

This may be important as the approximation (page 19 and 20) may implicitly assume that allele frequency and SNP variance are positively correlated, which is not the case for many traits. The scale factor, alpha, can be ranged from -2 to 2, in regardless of the number of QTLs.

3. I often see that key information is missing, e.g. In Figure 2, please add how to conduct repeated leaning? What is the sample sizes for the training and validation? The authors should carefully check and add elaborations if there is any missing information through the whole text.

4. What is dimension of x and y? what is dimension of x_j? what is dimension of x^Ty (line 18 in page 19)?

5. What is difference between X and x? Given the description in page 19 (line 3 - 5), they are the same. Please make this clear.

6. In Methods section, vector/matrix should be bold without italic so that readers can more easily understand the formulas.

7. At line 18 (page 19), E(XY) is a confusing term. What is the dimension of X? I thought it is N x # SNPs, but the term, XY, is not valid for this. Please clarify.

8. In the descriptions (pages 19 - 22), it is confusing which terms are from the full GWAS data and which terms are from subsamples (training or validation). The authors should elaborate or add notations to make those terms clearer.

9. In step 2, readers may want to see how accurate the approximated covariance estimates (line 17 and 18 in page 20). Can the authors plot or get a correlation coefficient between the sigma_j and sigma-hat_j, and sigma_ji and sigma-hat_ji?

Sigma_j and sigma_ji can be obtained from actual subsamples as described. Sigma-hat_j and sigma-hat_ji can be obtained from the formulas (line 10 and 11 in page 20), using the GWAS summary stats without subsampling. This validation may require multiple replicates with various values for heritability (e.g. 0.2 and 0.8 to check its robustness as the authors did for Figure 2). The authors should also consider varying samples sizes or other parameters (e.g. # QTLs) if they are a determining factor for the accuracy of the approximation.

10. Is the approximation still robust when causal (QTL) effects are inversely associated with MAF (in relation to the question #2)?

11. At line 21 in page 21, the second y should be y-hat?

12. In page 25 (GWAS data description), the authors should substantially revise and add detailed information so that readers should know what exactly have done. How many SNPs were used for the training and validation datasets? The authors only mentioned that they applied QC < 0.8 INFO scores for HRS samples, but no details for other GWAS data. It is not clear if the authors accessed individual genotypes for ADGC data (probably not). What is summary statistics-based R^2 and how did the authors get this values? The detail of UK Biobank genotypic data is totally missing.

13. Is theory and derivation valid for binary trait? I might miss, but I couldn't find any part to address this issue. Furthermore, I don't think the authors considered any binary trait in their simulation. I think the authors should verify their approach using binary phenotype simulations before applying it to AD as a real data analysis.

14. Given the description in page 24, PUMAS would perform 4-fold repeated learning. But, I thought PUMAS would not require such leave-one-out analyses. Please clarify this (also in relation to question #3).

15. Proper references should be added in Discussion, e.g. more sophisticated PRS methods have emerged, for which the authors should provide references.

16. The authors should explicitly discuss their limitations, e.g. 1) PUMAS would not be very useful for highly polygenic traits as shown in Figure 1A, 2) approximated R^2 can be less accurate for binary traits (or need to be verified in a further study) and 3) PUMAS was not tested for family-based GWAS that can be substantially efficient in polygenic risk prediction (see the following reference).

Efficient polygenic risk scores for biobank scale data by exploiting phenotypes from inferred relatives. Nature Communications volume 11, Article number: 3074 (2020)

**Reviewer 2**

**Were you able to assess all statistics in the manuscript, including the appropriateness of statistical tests used?** Yes

**Were you able to directly test the methods?** No

**Comments to author:**

Qiongshi Lu and colleagues present PUMAS, a method to fine-tune models that construct polygenic risk score (PRS) from association summary statistics from genome-wide association studies (GWAS). The authors propose an innovative approach to fine tune PRS from one set of summary statistics by "splitting" the data into training and validation. Such "splitting" is enabled by taking advantage of the analytical distribution of the association summary statistics, as detailed in their derivations. The approach is statistically elegant, and simple to implement. The authors conducted simulation studies and applied PUMAS to GWAS results of 65 different traits. The manuscript is well written with methods clearly presented, extensive real data analyses, and many important practical factors assessed. The authors also took one step further to associate the PRS derived into downstream association analysis. Specifically, they constructed PRS for brain imaging traits and associated these PRSs derived for brain imaging traits with Alzheimer's disease (AD) status, discovering two novel associations that seem sensible.

Overall, I am very enthusiastic about this manuscript. The PUMAS method presented will become a handy tool. I only have multiple minor comments.

First, the authors mentioned that PUMAS was computationally efficient but I didn't seem to find any statistics. Can the authors provide some more information regarding computational costs?

Second, the simulation studies, albeit comprehensive in terms of encompassing different sample sizes, overall trait heritability, and number of causal variants, considered only 5,000 variants in total (including causal and non-causal) and seems all at MAF of 20%. Although the method is theoretically accommodating LD, simulating genotypes of different but exchangeable markers from independent Binomail distribution with success probability of 0.2 without considering LD seems a bit overly simplistic.

Figure 3, AD pattern from both PUMAS and ADGC validation show some increasing pattern towards the very lenient end by including a lot of markers with large/insignificant p-values. Can the authors elaborate on that phenomenon?

Page 19, lines 21-22, the authors put forward the distributional property and say "It can be shown". Since this forms the foundation of the PUMAS work, I would like the authors to show the detailed derivations in supplemental materials.

**Authors Response**

**Point-by-point responses to the reviewers’ comments:**

**Reviewer #1:** 

**This is an interesting study proposing a novel method (PUMAS) to select an association p-value threshold for genome-wide SNPs that provides a better accuracy of polygenic risk prediction. To select an optimised p-value threshold, PUMA can obtain R^2 in a validation set, based on the conditional distribution of estimated SNP effects, which are derived from GWAS summary stats, without accessing the individual genotypes of the training dataset. The theoretical R^2 are evaluated to select an optimised p-value threshold that may result in the highest prediction accuracy. 

This is a neat and nice theoretical work. However, I have a lot of problems in reading the manuscript particularly for the derivation and description of the method (pages 19 - 22). The authors would have provided an evidence that their derived theory is valid, and approximations used in their derivation are robust, which is missing in the current manuscript. Discussion should be improved in general. I have a number of questions and comments.**          

**1. Method overview. Figure 1B can be improved, e.g. the authors can indicate the process includes to derive the conditional distribution of estimated SNP effects, given the GWAS summary stats, and simulate GWAS summary stats for any number of training and validation datasets. In the text (page 5), please add equation numbers (shown in Methods) for the first and second steps so that readers can easily understand what the steps are.**

Thank you for the suggestions. Following the reviewer’s suggestion, we have added equation numbers in the **Methods** section and highlighted important equations in the **Results** section on page 5 to help readers better understand our method. We have also updated **Figure 1** in the manuscript to indicate important steps for PUMAS to fine-tune PRS with summary data.

**2. In simulations, I would suggest varying the genetic architecture of trait, e.g. the causal effects are proportional to the minor allele frequencies (SNP effect* = SNP effect * (2p(1-p))^alpha where p is the reference allele frequency and alpha is the scale parameter to determine the genetic architecture. Please see equation 5 and Figure 2 in the following paper.**   

**Improved Heritability Estimation from Genome-wide SNPs. AJHG: 91: 1011. 

This may be important as the approximation (page 19 and 20) may implicitly assume that allele frequency and SNP variance are positively correlated, which is not the case for many traits. The scale factor, alpha, can be ranged from -2 to 2, in regardless of the number of QTLs.**

Thank you for the suggestions. First, we would like to clarify that our methodological framework does not make any assumptions on the distribution of SNP effects. On page 19 of our revised manuscript, we denote effect sizes $\boldsymbol{\beta}$ as unknown fixed quantities and $\hat{\boldsymbol{\beta}}$ as marginal regression coefficients estimated in GWAS, without assuming SNP effects to follow a particular distribution. However, the reviewer raised an important question about the robustness of PUMAS under different data generating models and our previous simulation setting might have been over-simplistic.

To better evaluate the performance of PUMAS, and also following Reviewer 2’s related comments on simulation settings, we have improved our simulation study using real genotype data from Wellcome Trust Case Control Consortium (WTCCC) under various genetic architecture. The WTCCC dataset contains 15,918 samples with 393,273 genotyped SNPs across the whole genome. We removed SNPs that are not available in 1000 Genomes Project Phase III European samples from the simulations since 1000 Genomes data were used as the LD reference panel (1). We excluded individuals with genotype missingness rate higher than 0.01 and removed SNPs that satisfy any of the following conditions: (i) having minor allele frequency less than 0.01, (ii) having missing rate higher than 0.01 among all subjects, and (iii) having p-value from the Hardy-Weinberg Equilibrium Test lower than 1e-6. After quality control, 322,235 variants and 15,567 samples remained in the analyses. We first simulated effect sizes $\beta_{j}$ from a normal distribution $N\left( 0,\frac{h^{2}}{Mp} \right)$ where $h^{2}$ is the heritability (fixed at 0.5), $M$ is the total number of SNPs, and $p$ is the proportion of causal variants. We chose two values of $p$ (i.e., 0.001 and 0.1) to represent sparser and more polygenic genetic architecture. Following the reviewer’s suggestion and the LDAK paper (2), we then replaced the raw effect sizes by $\beta_{j}^{*}=\beta_{j}\left[ 2p_{j}\left( 1-p_{j} \right) \right]^{\alpha}$ where $\alpha=-2,-1, 0, 1, 2$. Thus, in total we conducted simulations under 10 different settings. In each setting, causal SNPs were randomly selected across the genome and the effect sizes of non-causal SNPs were set to be 0. Using these simulated effect sizes, we generated continuous trait values in GCTA (3). We then performed marginal linear regressions and obtained GWAS summary statistics using PLINK (4). These summary statistics were used as input for PUMAS.

We compared PUMAS with repeated learning to demonstrate that PUMAS’s performance is comparable to methods that require individual-level data. Here, we implemented a 4-fold repeated learning approach (i.e., cross validation with replacement). In each fold, we randomly select 75% of WTCCC samples to perform GWAS, and evaluate the predictive performance of PRS on the remaining 25% of individuals. We repeated this process 4 times and reported the average predictive $R^{2}$ for each PRS model with different p-value cutoffs. For comparison, we implemented PUMAS in a similar fashion. Based on the GWAS summary data computed from all WTCCC samples, PUMAS generates a set of summary statistics for 75% of samples as the training data for PRS, and evaluates the predictive performance of PRS on the corresponding validation summary statistics (i.e., summary statistics for the remaining 25% of samples). We repeated this procedure 4 times and reported the average $R^{2}$ for each PRS model. In this simulation, we consider PRS models with p-value cutoffs ranging from 1e-5 to 1.

**Figure (Supplementary Figure 1)**. Comparison of two model-tuning strategies in WTCCC samples under alpha = -2. (A) PUMAS performance under a causal variant proportion of 0.001. (B) Repeated learning approach with individual-level data as input under a causal variant proportion of 0.001. (C) PUMAS performance under a causal variant proportion of 0.1. (D) Repeated learning approach with individual-level data as input under a causal variant proportion of 0.1. The X-axis shows the log-transformed p-value thresholds. The Y-axis shows the predictive performance quantified by average $R^{2}$ across four folds.

Here, we show that PUMAS performs as well as the repeated learning approach under new simulation settings. Overall, the optimal tuning parameter values (i.e., p-value cutoff) selected by PUMAS and repeated learning were very close in all settings. As shown in the figure above, when SNP effects are inversely proportional to allele frequency ($\alpha=-2$; i.e., rarer alleles tend to have larger effects), PUMAS and repeated learning selected identical p-value thresholds (i.e., 1e-5 and 1e-4 respectively) for two simulation settings with different numbers of causal variants. In the figure below, when true effect sizes are proportional to allele frequency ($\alpha=2$; i.e., rarer alleles tend to have weaker effects), both PUMAS and repeated learning selected 1e-4 as the optimal p-value cutoff when proportion of causal variant is 0.001 and suggested polygenic models when 10% of SNPs are causal. Although different assumptions about the relationship between SNP effects and allele frequency can lead to distinct optimal PRS models, PUMAS always shows consistent results compared to repeated learning.

**Figure (Supplementary Figure 4)**. Comparison of two model-tuning strategies in WTCCC samples under alpha = 2. (A) PUMAS performance under a causal variant proportion of 0.001. (B) Repeated learning approach with individual-level data as input under a causal variant proportion of 0.001. (C) PUMAS performance under a causal variant proportion of 0.1. (D) Repeated learning approach with individual-level data as input under a causal variant proportion of 0.1. The X-axis shows the log-transformed p-value thresholds. The Y-axis shows the predictive performance quantified by average $R^{2}$ across four folds.

We have rewritten the simulation sections in our manuscript following these new analyses. We have added the above figures as **Supplementary Figures 1** and **4**. We have replaced the original **Figure 2** with the simulation results when $\alpha=0$ and revised simulation results in the **Results** section on page 7 and simulation settings in the **Methods** section on page 25. Results for the remaining simulation settings have been added as **Supplementary Figures 2-3**. We have summarized each PRS’ predictive performance evaluated by PUMAS and repeated learning in every simulation setting in **Supplementary Table 1**.

**3. I often see that key information is missing, e.g. In Figure 2, please add how to conduct repeated leaning? What is the sample sizes for the training and validation? The authors should carefully check and add elaborations if there is any missing information through the whole text.**  

Thank you for your suggestion. The repeated learning approach we implemented in our analyses is also referred to as Monte Carlo cross-validation which is commonly used for machine learning model tuning. Instead of partitioning $N$ samples into $k$ non-overlapping folds, which is what a $k$-fold cross validation does, repeated learning randomly selects $\frac{N\left( k-1 \right)}{k}$ samples to form the training dataset and evaluates the model performance on the remaining $\frac{N}{k}$ samples. This procedure is then repeated $k$ times to obtain an averaged prediction accuracy (i.e., $R^{2}$) across $k$ folds of analysis for each prediction model. Therefore, in the new simulations using WTCCC genotype data, the size of training dataset $=\frac{3}{4}\times15567\approx11675$, and the size of validation dataset is $15567-11675= 3892$. We have added an explanation of how to conduct repeated learning and details of exact sample sizes for the training and validation dataset in each fold of analysis in the **Methods** section on page 25.

**4. What is dimension of x and y? what is dimension of x_j? what is dimension of x^Ty (line 18 in page 19)?** 

We use $N$ to denote the sample size and $p$ to denote the total number of SNPs in the GWAS data. Then, the dimension of the observed genotype and phenotype data, denoted as $\mathbf{x}=\left( \mathbf{x}_{1},\ldots,\mathbf{x}_{p} \right)$ and $\boldsymbol{y}$, are $N\times p$ and $N\times1$, respectively. Thus, the dimension of the observed genotype data for the $j$-th SNP, $\mathbf{x}_{j}$, is $N\times1$, and the dimension of summary statistics $\mathbf{x}^{T}\mathbf{y}$ is $p\times1$. We have added related explanation of these notations in the **Methods** section on page 19.

**5. What is difference between X and x? Given the description in page 19 (line 3 - 5), they are the same. Please make this clear.**

In the **Methods** section, $\mathbf{X}=\left( X_{1},\ldots,X_{p} \right)$ and $\mathbf{x}=\left( \mathbf{x}_{1},\ldots,\mathbf{x}_{p} \right)$ denote the random vector of genotypes and the observed genotype matrix at $p$ SNPs, respectively. They are conceptually similar but we use these notations to distinguish random variables from observed values. We have added related texts to describe the difference between $\mathbf{X}$ and $\mathbf{x}$ in the **Methods** section, on page 18.

**6. In Methods section, vector/matrix should be bold without italic so that readers can more easily understand the formulas.**  

Thank you very much for your suggestion. We have revised the text following this comment throughout the **Methods** section.

**7. At line 18 (page 19), E(XY) is a confusing term. What is the dimension of X? I thought it is N x # SNPs, but the term, XY, is not valid for this. Please clarify.**   

Thank you for your comment. We agree with the reviewer that our previous notation $E\left( \mathbf{X}Y \right)$ can be confusing. Following multiple comments on the notation, we have substantially improved the writing in our **Methods** section. We have updated this specific term into $E\left( \mathbf{X}^{T}Y \right)$, with $\mathbf{X}=\left( X_{1},\ldots,X_{p} \right)$ denoting the $1\times p$ random vector of genotype for $p$ SNPs. We have added more detailed description of notations in the **Methods** section, on page 20.

**8. In the descriptions (pages 19 - 22), it is confusing which terms are from the full GWAS data and which terms are from subsamples (training or validation). The authors should elaborate or add notations to make those terms clearer.**

Thank you very much for your suggestion. We only calculate ${\hat{\beta}_{j}}^{\left( tr \right)}$, $\mathrm{SE}\left( {\hat{\beta_{j}}}^{\left( tr \right)} \right)$, and correspondent p-value from the subsampled training summary statistics, ${\mathbf{x}^{\left( tr \right)}}^{\top}\mathbf{y}^{\left( tr \right)}$. The rest of estimators come from the full GWAS data or the external reference panel (i.e., 1000 Genomes Phase III European genotype data (1)), including the estimated SNP variance $\hat{\sigma}_{j}^{2}$ and approximated $\mathrm{Var}\left( Y \right)$ which is used for $R^{2}$ calculation. We have added related texts to help readers better understand different terms in the **Methods** section, on page 20.

**9. In step 2, readers may want to see how accurate the approximated covariance estimates (line 17 and 18 in page 20). Can the authors plot or get a correlation coefficient between the sigma_j and sigma-hat_j, and sigma_ji and sigma-hat_ji? 

Sigma_j and sigma_ji can be obtained from actual subsamples as described. Sigma-hat_j and sigma-hat_ji can be obtained from the formulas (line 10 and 11 in page 20), using the GWAS summary stats without subsampling. This validation may require multiple replicates with various values for heritability (e.g. 0.2 and 0.8 to check its robustness as the authors did for Figure 2). The authors should also consider varying samples sizes or other parameters (e.g. # QTLs) if they are a determining factor for the accuracy of the approximation.** 

This is a great suggestion and we really appreciate it. To investigate how accurate our approximated covariance for $\mathbf{x}^{T}\mathbf{y}$ is, we conducted additional simulations on the WTCCC genotype data. Following the reviewer’s suggestion, we used a total of 8 different simulation settings with sample size $N$ of 5000 and 15567, heritability $h^{2}$ of 0.2 and 0.8, and proportion of causal variants being 0.001 and 0.1. We used $\alpha=2$ in all settings to transform raw effect size to true SNP effects following reviewer’s comment #2 such that causal effects are inversely associated with allele frequencies. We simulated quantitative trait values and obtained summary statistics from marginal linear regressions.

Then, we calculated and compared diagonal elements $\Sigma_{j}$ and $\hat{\Sigma}_{j}$, and off-diagonal elements $\Sigma_{ij}$ and $\hat{\Sigma}_{ij}$, respectively. Note that in our method, theoretical values of elements in the covariance matrix are

$$\boldsymbol{\Sigma}_{j}=\beta_{j}^{2}\mathrm{Var}\left( X_{j}^{2} \right)+E\left( \epsilon_{j}^{2} \right)E\left( X_{j}^{2} \right)$$

$$\boldsymbol{\Sigma}_{ji}=\beta_{j}\beta_{i}E\left( X_{j}^{2} \right)E\left( X_{i}^{2} \right)$$

We calculated $\mathrm{Var}\left( X_{j}^{2} \right)$ by the empirical variance of squared genotype data. $E\left( X_{j}^{2} \right)$ and $E\left( X_{i}^{2} \right)$ were computed using the empirical mean of squared genotype data. We calculated $E\left( \epsilon_{j}^{2} \right)$, the expectation of squared residual in the marginal linear regression for the $j$-th SNP by

$$E\left( \epsilon_{j}^{2} \right)=Var\left( Y-\beta_{j}X_{j} \right)=Var\left( Y \right)-\beta_{j}^{2}Var\left( X_{j} \right)$$

where the second equation holds when SNPs are independent, which is true since the GWAS summary statistics were LD-pruned in advance. We computed $Var\left( Y \right)$ and $Var\left( X_{j} \right)$ by the empirical variance of phenotype and genotype data of the $j$-th variant. Then we obtain our framework’s approximation of the covariance matrix by

$${\hat{\boldsymbol{\Sigma}}}_{j}=N\left[ \mathrm{SE}\left( \hat{\beta}_{j} \right)\hat{\sigma}_{j}^{2} \right]^{2}$$

$${\hat{\boldsymbol{\Sigma}}}_{ji}=\hat{\beta}_{j}\hat{\beta}_{i}\hat{\sigma}_{j}^{2}\hat{\sigma}_{i}^{2}$$

where $\hat{\beta}_{j}$ and $\mathrm{SE}\left( \hat{\beta}_{j} \right)$ are the regression coefficient and standard error from the marginal linear model of the $j$-th SNP. $\hat{\sigma}_{j}^{2}$ is the estimator of SNP variance using minor allele frequency from WTCCC data.

It is straightforward to compare the diagonal elements since the number of SNPs remained after LD-pruning for all settings were around 50,000. However, it becomes computationally intensive if we compare all off-diagonal elements at once since the length of this vector containing all off-diagonal terms would reach the size of 10^9^. Therefore, in each setting we randomly picked off-diagonal elements from the covariance matrix such that the length of this vector is equivalent to the number of diagonal terms in the covariance matrix. We then plotted both $\hat{\Sigma}_{j}$ and $\hat{\Sigma}_{ij}$ against $\Sigma_{j}$ and $\Sigma_{ij}$ and calculate their pairwise correlations. As shown in the below table, the correlations between $\hat{\Sigma}_{j}$ and $\Sigma_{j}$ are always higher than 0.99 in all of 8 simulation settings; on the other hand, the correlations between $\hat{\Sigma}_{ij}$ and $\Sigma_{ij}$ are low (the occurrence of NA is due to the fact that most SNPs in the genetic setting with proportion of causal variants=0.001 have true effects of 0 and the randomly selected off-diagonal terms $\Sigma_{ij}$ are all 0). However, we note that the low correlation between $\hat{\Sigma}_{ij}$ and $\Sigma_{ij}$ is due to the extremely small scale of off-diagonal elements in the covariance matrix. In fact, as shown in the figure below, when we plotted all diagonal and randomly selected off-diagonal elements in the same figure, the off-diagonal elements are much smaller than diagonal terms in all settings. This suggests that off-diagonal terms have almost no effect when we subsample summary statistics from the conditional distribution ${\mathbf{x}^{\left( tr \right)}}^{\top}\mathbf{y}^{(tr)}\mid\mathbf{x}^{\top}\mathbf{y}$ in practice. This observation is consistent with our current implementation of PUMAS that we did not consider the off-diagonal terms when generating training and testing summary statistics given the full GWAS summary data, which greatly boosts computational speed yet maintains the accuracy of the subsampled summary statistics. We have added related discussion in the **Methods** section on page 26. We have also included the following table and figure as **Supplementary Table 12** and **Supplementary Figure 15**.

| Setting | N | h2 | p | alpha | diagonal cor | off-diagonal cor |
| --- | --- | --- | --- | --- | --- | --- |
| 1 | 15567 | 0.2 | 0.001 | 2 | 0.9993 | 0.2866 |
| 2 | 15567 | 0.2 | 0.1 | 2 | 0.9993 | 0.0090 |
| 3 | 15567 | 0.8 | 0.001 | 2 | 0.9993 | NA |
| 4 | 15567 | 0.8 | 0.1 | 2 | 0.9993 | 0.0477 |
| 5 | 5000 | 0.2 | 0.001 | 2 | 0.9982 | NA |
| 6 | 5000 | 0.2 | 0.1 | 2 | 0.9982 | 0.0101 |
| 7 | 5000 | 0.8 | 0.001 | 2 | 0.9982 | NA |
| 8 | 5000 | 0.8 | 0.1 | 2 | 0.9982 | 0.0162 |

**Table (Supplementary Table 12)**. Simulation settings for investigating the estimation accuracy of covariance of $\mathbf{x}^{\top}\mathbf{y}$.

**Figure (Supplementary Figure 15)**. Comparison of PUMAS’s approximated $\Sigma$ and theoretical $\Sigma$ in 8 simulation settings. (A-H) scatter plots of approximated diagonal and off-diagonal elements versus theoretical diagonal and off-diagonal elements in each setting. Details on simulations settings are discussed in the **Methods** section.

**10. Is the approximation still robust when causal (QTL) effects are inversely associated with MAF (in relation to the question #2)?**

In our response to comment #9, we have conducted simulation studies under the setting where causal effects are inversely associated with minor allele frequencies. As discussed above, we found that our proposed framework’s approximation of the covariance matrix for $\mathbf{x}^{T}\mathbf{y}$ is accurate.

**11. At line 21 in page 21, the second y should be y-hat?** 

Thank you for the comment. This is indeed a typo. We have changed $\bar{\mathbf{y}^{\left( v \right)}}$ to $\bar{{\hat{\mathbf{y}}}^{\left( v \right)}}$ on page 22.

**12. In page 25 (GWAS data description), the authors should substantially revise and add detailed information so that readers should know what exactly have done. How many SNPs were used for the training and validation datasets? The authors only mentioned that they applied QC < 0.8 INFO scores for HRS samples, but no details for other GWAS data. It is not clear if the authors accessed individual genotypes for ADGC data (probably not). What is summary statistics-based R^2 and how did the authors get this values? The detail of UK Biobank genotypic data is totally missing.**

Thank you for your suggestion. We provide details on these datasets below and have added these details into the **Methods** section of our revised manuscript.

GWAS summary statistics for educational attainment (EA (5)) and Alzheimer’s disease (AD (6)) include 10,824,042 and 7,055,881 SNPs, respectively; after LD-pruning, 104,526 and 203,118 SNPs remained in the data. For the analysis of 65 complex traits, we have summarized the number of SNPs in each file in **Supplementary Table 6**.

The Add Health genotype data were processed and imputed by the Add Health analysis team. The comprehensive data cleaning procedure is documented on the Add Health website at https://addhealth.cpc.unc.edu/wp-content/uploads/docs/user_guides/AH_GWAS_QC.pdf. The genotype data were imputed using 1000 Genomes Project Phase III European and non-European samples. The final Add Health genotype data contains 9,974 samples and 9,664,514 SNPs in total. Of these 9,974 samples, we found phenotype information on 4,775 European samples and used them for the EA analysis. The HRS dataset was also pre-processed and imputed to the 1000 Genomes reference. The imputed genotype data has 15,567 samples and 18,144,468 SNPs. We applied a threshold of 0.8 on the imputation $R^{2}$ to obtain the final genotype data used for EA analysis. We matched 10,214 samples with phenotype information and used these samples for the analysis on EA. The UK Biobank genotype data was imputed to the Haplotype Reference Consortium (HRC) reference. We removed samples who are not of European ancestry and SNPs with minor allele frequency < 0.01 or imputation $R^{2}$ < 0.9. The final UKB genotypic data has 9,605,099 SNPs for 355,583 individuals.

We did not obtain individual-level genotype and phenotype data from ADGC. Instead, we used the ADGC GWAS summary statistics (number of SNPs=9,037,014) of 7050 independent samples (7) not included in the IGAP 2013 GWAS as the testing summary statistics to evaluate the predictive performance of AD PRS. The summary-statistics-based $R^{2}$ was calculated exactly following our proposed method (**Methods**, equation (20) on page 23). In most PUMAS applications, we first subsample training and testing GWAS summary statistics, and then calculate $R^{2}$ on the testing summary statistics. In this case, since we have a set of independent testing summary statistics from ADGC, we skipped subsampling and treated IGAP 2013 AD GWAS as the training summary statistics and ADGC GWAS as the testing summary statistics.

**13. Is theory and derivation valid for binary trait? I might miss, but I couldn't find any part to address this issue. Furthermore, I don't think the authors considered any binary trait in their simulation. I think the authors should verify their approach using binary phenotype simulations before applying it to AD as a real data analysis.**     

This is a great point. The derivations in our original submission were based on quantitative traits. To show that PUMAS can be applied to binary traits, we conducted additional simulations under settings described in our response to comment #2. For each simulation setting, we kept the same SNP effects, heritability, and proportion of causal variants. However, instead of generating quantitative phenotypes, we simulated binary phenotypes using a population prevalence of 50% and case-control ratio of 1:1 in GCTA (3). We performed marginal logistic regressions in PLINK (4) to obtain GWAS summary statistics. Like the simulation for quantitative traits, we compared the performance between PUMAS and 4-fold repeated learning using individual-level data for binary traits. For repeated learning, each time we randomly selected 75% samples from the WTCCC dataset to train the marginal logistic regression model and evaluated the predictive performance of PRS on the remaining 25% individuals. We repeated this process 4 times and reported the average prediction accuracy for each PRS model with different p-value cutoffs. We used area under the ROC curve (AUC) as the metric to assess prediction accuracy in repeated learning. For comparison, we implemented PUMAS in the fashion of 4-fold repeated learning as well: each time PUMAS generates the summary statistics for 75% of all GWAS samples as the training dataset and evaluates the predictive performance of PRS on the validation summary statistics for the remaining 25% of samples. We repeated this procedure 4 times, and reported the average predictive $R^{2}$ for each PRS model. In this simulation, we consider PRS models with p-value cutoffs ranging from 1e-5 to 1.

We found that PUMAS’ results are highly consistent with repeated learning in terms of the selected optimal tuning parameters. As shown in the figure below, when raw SNP effects are not further adjusted by allele frequency ($\alpha=0$), PUMAS and repeated learning selected 5e-5 and 5e-4 as the optimal p-value thresholds when the proportion of causal variant is 0.001, and selected p-value cutoffs of 0.7 and 0.8 when 10% of SNPs are causal. Even though PUMAS uses $R^{2}$ to quantify prediction accuracy, the trend of $R^{2}$ against different tuning parameters is concordant with AUC values computed in repeated learning with individual-level data, making PUMAS capable of tuning PRS model for binary traits. For the remaining settings with different assumptions of the relationship between effect sizes and allele frequency, the optimal tuning parameters of PRS evaluated by PUMAS and repeated learning are very close as well.

**Figure** (**Supplementary Figure 7**). Comparison of two model-tuning strategies for binary traits in WTCCC samples under alpha = 0. (A) PUMAS performance under a causal variant proportion of 0.001. (B) Repeated learning approach with individual-level data as input under a causal variant proportion of 0.001. (C) PUMAS performance under a causal variant proportion of 0.1. (D) Repeated learning approach with individual-level data as input under a causal variant proportion of 0.1. The X-axis shows the log-transformed p-value thresholds. The Y-axis shows the predictive performance quantified by average $R^{2}$ for PUMAS and AUC for repeated learning across four folds.

All simulation results for binary traits have been added as **Supplementary Figures 5-9**. We have summarized each PRS’ predictive performance evaluated by PUMAS and repeated learning in every simulation setting for binary traits in **Supplementary Table 2**. We have also added related discussion in the **Results** and **Methods** sections on page 7 and page 26.

**14. Given the description in page 24, PUMAS would perform 4-fold repeated learning. But, I thought PUMAS would not require such leave-one-out analyses. Please clarify this (also in relation to question #3).**

Thank you for your comment. We have discussed how PUMAS can implement repeated-learning-like approaches to fine-tune PRS models in response to comment #2 and how classic repeated learning based on individual-level data is implemented in response to comment #3. The key difference between leave-one-out analyses implemented in PUMAS and traditional model-tuning approaches is that PUMAS does not require individual-level data. Since PUMAS can subsample summary statistics for a subset of samples of the full GWAS summary data, it becomes a flexible framework that can utilize various model-tuning techniques to optimize PRS. For example, PUMAS can first generate summary statistics to train PRS for a half of total samples and evaluate predictive performance using summary statistics of the other half of samples. In this case, PUMAS implements a simple training/validation split to fine-tune PRS. Alternatively, PUMAS can generate summary statistics for 25% of samples for 3 times, and subsequently calculate summary statistics for the remaining 25% of samples. Then PUMAS is able to implement a 4-fold cross-validation by combining 3 separate summary statistics for 25% of samples to form the training dataset in each fold. If PUMAS simulates training summary statistics for 75% of samples for 4 times with replacement, the model-tuning approach used by PUMAS becomes repeated learning. We have highlighted this feature of PUMAS in the **Methods** section on page 24.

**15. Proper references should be added in Discussion, e.g. more sophisticated PRS methods have emerged, for which the authors should provide references.**  

Thank you for your suggestion. We have added citations and discussions for the following recent PRS methods in the **Discussion** section on page 18.

LDpred2: better, faster, stronger. Bioinformatics, Volume 36, Issue 22-23, 1 December 2020, Pages 5424–5431

A Penalized Regression Framework for Building Polygenic Risk Models Based on Summary Statistics From Genome-Wide Association Studies and Incorporating External Information. Journal of the American Statistical Association, Volume 116, 2021 - Issue 533

Polygenic prediction via Bayesian regression and continuous shrinkage priors. Nature Communications volume 10, Article number: 1776 (2019)

Accurate and Scalable Construction of Polygenic Scores in Large Biobank Data Sets. American Journal of Human Genetics, Volume 106, Issue 5, 7 May 2020, Pages 679-693

Improved polygenic prediction by Bayesian multiple regression on summary statistics. Nature Communications volume 10, Article number: 5086 (2019)

**16. The authors should explicitly discuss their limitations, e.g. 1) PUMAS would not be very useful for highly polygenic traits as shown in Figure 1A, 2) approximated R^2 can be less accurate for binary traits (or need to be verified in a further study) and 3) PUMAS was not tested for family-based GWAS that can be substantially efficient in polygenic risk prediction (see the following reference). 

Efficient polygenic risk scores for biobank scale data by exploiting phenotypes from inferred relatives. Nature Communications volume 11, Article number: 3074 (2020)** 

Thank you for your comments and suggestions. Regarding the first comment “**1) PUMAS would not be very useful for highly polygenic traits as shown in Figure 1A**”, we would like to point out that **Figure 1A** (in our original submission) does not imply that PUMAS is more useful for sparser traits compared to polygenic traits. In fact, PUMAS is a PRS model-tuning method based on GWAS summary statistics that can be used to determine whether the optimal PRS model is sparse or polygenic given the GWAS summary statistics. Without PUMAS, researchers must rely on external individual-level dataset to learn the optimal tuning parameter. If they do not have access to such data, the optimal PRS model remains unknown. Regarding the second comment “**2) approximated R^2 can be less accurate for binary traits (or need to be verified in a further study)**”, we agree that approximated $R^{2}$ itself is less interpretable for binary traits since PRS for binary traits are usually evaluated using AUC. However, as we have demonstrated in response to comment #12 that PUMAS’s approximated $R^{2}$ is consistent with AUC calculated in repeated learning with individual-level data, the approximated $R^{2}$ remains useful for selecting the optimal tuning parameter for PRS of binary traits. For “**3) PUMAS was not tested for family-based GWAS that can be substantially efficient in polygenic risk prediction**”, we agree with the reviewer that current framework of PUMAS is not generalized for family-based GWAS. We have included points 2) and 3) from the reviewer and added related discussion of these points in the **Discussion** section on page 18.

**Reviewer #2:**

**Qiongshi Lu and colleagues present PUMAS, a method to fine-tune models that construct polygenic risk score (PRS) from association summary statistics from genome-wide association studies (GWAS). The authors propose an innovative approach to fine tune PRS from one set of summary statistics by "splitting" the data into training and validation. Such "splitting" is enabled by taking advantage of the analytical distribution of the association summary statistics, as detailed in their derivations. The approach is statistically elegant, and simple to implement. The authors conducted simulation studies and applied PUMAS to GWAS results of 65 different traits. The manuscript is well written with methods clearly presented, extensive real data analyses, and many important practical factors assessed. The authors also took one step further to associate the PRS derived into downstream association analysis. Specifically, they constructed PRS for brain imaging traits and
associated these PRSs derived for brain imaging traits with Alzheimer's disease (AD) status, discovering two novel associations that seem sensible.

Overall, I am very enthusiastic about this manuscript. The PUMAS method presented will become a handy tool. I only have multiple minor comments.

1. First, the authors mentioned that PUMAS was computationally efficient but I didn't seem to find any statistics. Can the authors provide some more information regarding computational costs?**

Thank you for your positive and constructive comments. To quantify the computational efficiency of PUMAS, we repeated the analysis of 65 publicly available GWAS traits and recorded the elapsed time PUMAS used to complete each analysis. Then, we compared the computation time with number of SNPs in each LD-pruned GWAS summary statistics. As shown in the figure below, the time PUMAS spent on each trait increases as the number of SNPs increases. Using one CPU, the average computation time for 65 traits was 8.29 seconds and the maximum computation time was 38.23 seconds. These results demonstrate that PUMAS is computationally fast and efficient for analyzing real-world GWAS summary data.

**Figure (Supplementary Figure 12)**. Computation time for the analysis of 65 GWAS traits. The X-axis shows the number of SNPs in the pruned GWAS. The Y-axis shows the elapsed computation time in seconds.

We have included the above figure as **Supplementary Figure 12** and summarized the computation time and number of SNPs for 65 GWAS traits that we have analyzed in **Supplementary Table 8**. We have added related discussion in the **Results** section on page 15.

**2. Second, the simulation studies, albeit comprehensive in terms of encompassing different sample sizes, overall trait heritability, and number of causal variants, considered only 5,000 variants in total (including causal and non-causal) and seems all at MAF of 20%. Although the method is theoretically accommodating LD, simulating genotypes of different but exchangeable markers from independent Binomail distribution with success probability of 0.2 without considering LD seems a bit overly simplistic.**

Thank you for your comments and suggestions.	Following this comment and a related comment from Reviewer 1, we conducted additional simulations using real genotype data from the Wellcome Trust Case Control Consortium (WTCCC). The WTCCC dataset contains 15,918 samples with 393,273 genotyped SNPs across the whole genome. We removed SNPs that are not available in 1000 Genomes Project Phase III European samples from the simulations since 1000 Genomes data were used as the LD reference panel (1). We excluded individuals with genotype missingness rate higher than 0.01 and removed SNPs that satisfy any of the following conditions: (i) having minor allele frequency less than 0.01, (ii) having missing rate higher than 0.01 among all subjects, and (iii) having p-value from the Hardy-Weinberg Equilibrium Test lower than 1e-6. After quality control, 322,235 variants and 15,567 samples remained in the analyses. We first simulated effect sizes $\beta_{j}$ from a normal distribution $N\left( 0,\frac{h^{2}}{Mp} \right)$ where $h^{2}$ is the heritability (fixed at 0.5), $M$ is the total number of SNPs, and $p$ is the proportion of causal variants. We chose two values of $p$ (i.e., 0.001 and 0.1) to represent sparser and more polygenic genetic architecture. Following Reviewer 1’s suggestion and the LDAK paper (2), we then replaced the raw effect sizes by $\beta_{j}^{*}=\beta_{j}\left[ 2p_{j}\left( 1-p_{j} \right) \right]^{\alpha}$ where $\alpha=-2,-1, 0, 1, 2$ to better evaluate the performance of PUMAS under various genetic architecture. Thus, in total we conducted simulations under 10 different settings. In each setting, causal SNPs were randomly selected across the genome and the effect sizes of non-causal SNPs were set to be 0. Using these simulated effect sizes, we generated continuous trait values in GCTA (3). We then performed marginal linear regressions and obtained GWAS summary statistics using PLINK (4). These summary statistics were used as input for PUMAS.

We compared PUMAS with repeated learning to demonstrate that PUMAS’s performance is comparable to methods that require individual-level data. Here, we implemented a 4-fold repeated learning approach (i.e., cross validation with replacement). In each fold, we randomly select 75% of WTCCC samples to perform GWAS, and evaluate the predictive performance of PRS on the remaining 25% of individuals. We repeated this process 4 times and reported the average predictive $R^{2}$ for each PRS model with different p-value cutoffs. For comparison, we implemented PUMAS in a similar fashion. Based on the GWAS summary data computed from all WTCCC samples, PUMAS generates a set of summary statistics for 75% of samples as the training data for PRS, and evaluates the predictive performance of PRS on the corresponding validation summary statistics (i.e., summary statistics for the remaining 25% of samples). We repeated this procedure 4 times and reported the average $R^{2}$ for each PRS model. In this simulation, we consider PRS models with p-value cutoffs ranging from 1e-5 to 1.

Here, we show that PUMAS performs as well as the repeated learning approach under new simulation settings. Overall, the optimal tuning parameter values (i.e., p-value cutoff) selected by PUMAS and repeated learning were very close in all settings. As shown in the figure below, when SNP effects are not further adjusted ($\alpha=0$), both PUMAS and repeated learning selected 5e-5 as the optimal p-value cutoff when proportion of causal variant is 0.001 and suggested polygenic models when 10% of SNPs are causal. Although different assumptions about the relationship between SNP effects and allele frequency can lead to distinct optimal PRS models in the remaining settings, PUMAS always shows consistent results compared to repeated learning.

**Figure (Figure 2)**. Comparison of PUMAS and repeated learning. Panels **A** and **C** illustrate model tuning results based on PUMAS. Panels **B** and **D** show results of repeated learning with individual-level data as input. Proportion of causal variants was set to be 0.001 in **A-B** and 0.1 in **C-D**. The X-axis shows the log-transformed p-value thresholds. The Y-axis shows the predictive performance quantified by average $R^{2}$ across four folds. Parameter $\alpha$ was set to be 0 in this simulation (**Methods**).

We have rewritten the simulation sections in our manuscript following these new analyses. We have replaced the original **Figure 2** with the above figure for simulation results when $\alpha=0$ and revised simulation results in the **Results** section on page 7 and simulation settings in the **Methods** section on page 25. Results for the remaining simulation settings have been added as **Supplementary Figures 1-4**. We have summarized each PRS’ predictive performance evaluated by PUMAS and repeated learning in every simulation setting in **Supplementary Table 1**.

**3. Figure 3, AD pattern from both PUMAS and ADGC validation show some increasing pattern towards the very lenient end by including a lot of markers with large/insignificant p-values. Can the authors elaborate on that phenomenon?**  

Thank you for your comment. Following the reviewer’s question, we replicated the results for Alzheimer’s disease PRS in **Figure 3** and found that the increasing $R^{2}$ pattern at the end of the plot may be due to suboptimal GWAS data processing in the previous analysis. Since the IGAP 2013 AD GWAS summary statistics (6), which we applied PUMAS on, does not provide information about minor allele frequency, we had to obtain MAF from an external reference panel (i.e., 1000 Genomes European samples (1)). In our previous analysis, we removed SNPs in strong LD first and then obtained MAF from the reference panel. Such a procedure is not ideal because during MAF imputation, a number of SNPs were removed from the GWAS summary statistics since they did not exist in the external LD reference. In the revised manuscript, we have improved the analysis by imputing MAF at the beginning for the full AD GWAS summary data and obtaining the LD-pruned GWAS data afterwards. The number of SNPs in the analysis increased from 177,188 to 203,118. We applied PUMAS to the better-processed GWAS data and used ADGC summary statistics (7) to compute the out-of-sample predictive $R^{2}$. As shown in the figure below, PUMAS’ performance remains consistent with the ADGC external validation. Notably, the upward tail in both panels has disappeared. We have updated **Figure 3**, **Figure 4**, and **Supplementary Table 3** in the manuscript.

**Figure**. Model tuning performance on real Alzheimer’s disease GWAS. (A) PUMAS performance on the IGAP 2013 GWAS summary statistics (B) Out-of-sample prediction performance using IGAP 2019 GWAS as the training set and ADGC GWAS as the testing set.

**4. Page 19, lines 21-22, the authors put forward the distributional property and say "It can be shown". Since this forms the foundation of the PUMAS work, I would like the authors to show the detailed derivations in supplemental materials.**

Thank you very much for your suggestion. We have added the detailed derivation in **Supplementary Notes**. Here, we provide the derivation of the distribution of $\mathbf{x}^{(tr)^{T}}\mathbf{y}^{\left( tr \right)}|\mathbf{x}^{T}\mathbf{y}$ as the following:

We derive the distribution of $\mathbf{x}^{(tr)^{T}}\mathbf{y}^{\left( tr \right)}|\mathbf{x}^{T}\mathbf{y}$ using the formula for conditional distribution of two multivariate normal random vectors. For example, if $\mathbf{X}\sim N\left( \boldsymbol{\mu}_{\mathbf{X}},\boldsymbol{\Sigma}_{\mathbf{X}} \right)$ and $\mathbf{Y}\sim N\left( \boldsymbol{\mu}_{\mathbf{Y}}, \boldsymbol{\Sigma}_{\mathbf{Y}} \right)$, and $cov\left( \mathbf{X},\mathbf{Y} \right)=\boldsymbol{\Sigma}_{\mathbf{XY}}$, then the distribution of $\mathbf{X}|\mathbf{Y}$ also follows a multivariate normal distribution with mean and covariance matrix

$$E\left( \mathbf{X} | \mathbf{Y}=\mathbf{y} \right)=\boldsymbol{\mu}_{\mathbf{X}}+\boldsymbol{\Sigma}_{\mathbf{XY}}\boldsymbol{\Sigma}_{\mathbf{Y}}^{-1}\left( \mathbf{y}-\boldsymbol{\mu}_{\mathbf{Y}} \right)$$

$$Var\left( \mathbf{X} | \mathbf{Y}=\mathbf{y} \right)=\boldsymbol{\Sigma}_{\mathbf{X}}-\boldsymbol{\Sigma}_{\mathbf{XY}} \boldsymbol{\Sigma}_{\mathbf{Y}}^{-1} \boldsymbol{\Sigma}_{\mathbf{YX}}$$

Based on this property, since

$$\mathbf{x}^{T}\mathbf{y}\sim N(NE(\mathbf{X}^{T}Y),NVar(\mathbf{X}^{T}Y))$$

$$\mathbf{x}^{(tr)^{T}}\mathbf{y}^{(tr)}\sim N((N-n)E(\mathbf{X}^{T}Y),(N-n)Var(\mathbf{X}^{T}Y))$$

and the covariance between $\mathbf{x}^{(tr)^{T}}\mathbf{y}^{(tr)}$ and $\mathbf{x}^{T}\mathbf{y}$ is

$$\begin{matrix} cov(\mathbf{x}^{(tr)^{T}}\mathbf{y}^{(tr)},\mathbf{x}^{T}\mathbf{y}) & =cov(\mathbf{x}^{(tr)^{T}}\mathbf{y}^{(tr)},\mathbf{x}^{(tr)^{T}}\mathbf{y}^{(tr)}+\mathbf{x}^{(v)^{T}}\mathbf{y}^{(v)}) \\ & =Var(\mathbf{x}^{(tr)^{T}}\mathbf{y}^{(tr)}) \\ & =(N-n)Var(\mathbf{X}^{T}Y) \end{matrix}$$

Then we can write the expectation of the distribution of $\mathbf{x}^{(tr)^{T}}\mathbf{y}^{\left( tr \right)}|\mathbf{x}^{T}\mathbf{y}$ as

$$E(\mathbf{x}^{(tr)^{T}}\mathbf{y}^{(tr)}|\mathbf{x}^{T}\mathbf{y})=(N-n)E(\mathbf{X}^{T}Y)+\frac{N-n}{N}Var(\mathbf{X}^{T}Y)Var(\mathbf{X}^{T}Y)^{-1}(\mathbf{x}^{T}\mathbf{y}-NE(\mathbf{X}^{T}Y))$$

Replace $NE(\mathbf{X}^{T}Y)$ by the observed vector $\mathbf{x}^{T}\mathbf{y}$, we get

$$E(\mathbf{x}^{(tr)^{T}}\mathbf{y}^{(tr)}|\mathbf{x}^{T}\mathbf{y})=\frac{N-n}{N}\mathbf{x}^{T}\mathbf{y}$$

And the variance of the conditional distribution is

$$\begin{matrix} Var(\mathbf{x}^{(tr)^{T}}\mathbf{y}^{(tr)}|\mathbf{x}^{T}\mathbf{y}) & =(N-n)Var(\mathbf{X}^{T}Y)-\frac{N-n}{N}Var(\mathbf{X}^{T}Y)Var(\mathbf{X}^{T}Y)^{-1}(N-n)Var(\mathbf{X}^{T}Y) \\ & =\frac{(N-n)n}{N}Var(\mathbf{X}^{T}Y) \end{matrix}$$

Replace $Var\left( \mathbf{X}^{T}Y \right)$ by the observed covariance matrix $\boldsymbol{\Sigma}$, then

$$Var\left( \mathbf{x}^{(tr)^{T}}\mathbf{y}^{\left( tr \right)} | \mathbf{x}^{T}\mathbf{y} \right)=\frac{\left( N-n \right)n}{N} \boldsymbol{\Sigma}$$

**References**

1.	McVean GA, Altshuler DM, Durbin RM, Abecasis GR, Bentley DR, Chakravarti A, et al. An integrated map of genetic variation from 1,092 human genomes. Nature. 2012;491(7422):56-65.

2.	Speed D, Hemani G, Johnson MR, Balding DJ. Improved heritability estimation from genome-wide SNPs. American journal of human genetics. 2012;91(6):1011-21.

3.	Yang J, Lee SH, Goddard ME, Visscher PM. GCTA: a tool for genome-wide complex trait analysis. Am J Hum Genet. 2011;88(1):76-82.

4.	Purcell S, Neale B, Todd-Brown K, Thomas L, Ferreira MA, Bender D, et al. PLINK: a tool set for whole-genome association and population-based linkage analyses. Am J Hum Genet. 2007;81(3):559-75.

5.	Lee JJ, Wedow R, Okbay A, Kong E, Maghzian O, Zacher M, et al. Gene discovery and polygenic prediction from a genome-wide association study of educational attainment in 1.1 million individuals. Nat Genet. 2018;50(8):1112-21.

6.	Lambert JC, Ibrahim-Verbaas CA, Harold D, Naj AC, Sims R, Bellenguez C, et al. Meta-analysis of 74,046 individuals identifies 11 new susceptibility loci for Alzheimer's disease. Nat Genet. 2013;45(12):1452-8.

7.	Hu Y, Li M, Lu Q, Weng H, Wang J, Zekavat SM, et al. A statistical framework for cross-tissue transcriptome-wide association analysis. Nature Genetics. 2019;51(3):568-76.

**Second round of review**

**Reviewer 1**

The manuscript has been significantly improved. The authors have addressed most of my concerns for the previous version. I have no further comments.

**Reviewer 2**

The authors have addressed my comments carefully and I have no additional comments.
